# A user-friendly strategy to engineer tailored intermediate strains for overcoming combined type I and type II restriction-modification barriers in *Staphylococcus aureus*

**DOI:** 10.1016/j.synbio.2026.05.016

**Published:** 2026-07-03

**Authors:** Yang Zheng, Chao Li, Xinqi Huang, Xinyi Shou, Runzhe Su, Jinyao Zhang, Qiwen Hu, Weilong Shang, Xiancai Rao, Renjie Zhou, Xiao-Ran Jiang

**Affiliations:** a Department of Emergency Medicine, the Second Affiliated Hospital of Army Medical University, Chongqing, 400037, China; b Key Laboratory of Microbial Engineering Under the Educational Committee in Chongqing, Department of Microbiology, College of Basic Medical Sciences, Army Medical University, Chongqing, 400038, China

**Keywords:** *Staphylococcus aureus*, Restriction-modification systems, Plasmid transformation, Intermediate strains, Clinical isolates

## Abstract

A significant proportion of *Staphylococcus aureus* clinical isolates are refractory to plasmid transformation due to the combined action of type I and type II restriction-modification (RM) systems. Current strategies often depend on specialized engineered strains and may require sequence modifications if foreign DNA harbors host-specific methylation patterns, limiting their general applicability. Here, we developed a user-friendly, sequence-independent strategy that employs customized restriction-deficient intermediate hosts. Using the sequence type 121 (ST121) strain XQ as a proof-of-concept, we first engineered an *Escherichia coli* strain to produce the requisite type I methylation pattern, enabling low-efficiency plasmid transformation. These plasmids were then used to delete the type I and type II restriction genes in XQ, creating the highly transformable intermediate strain XQ01. Once constructed, even plasmids prepared from common *E. coli* cloning hosts (e.g., TOP10) could be consistently transformed into XQ01. Plasmids propagated in XQ01 were subsequently transformed into wild-type XQ and other ST121 clinical isolates with high efficiency (>10^4^ transformants per μg DNA). This streamlined approach allowed us to successfully generate knockout, insertion, and fusion-expression mutants in XQ or its variants. Collectively, this work establishes a versatile transformation strategy that effectively bypasses type I and II RM barriers, facilitating genetic manipulation of ST121 and other RM-harboring *S. aureus* strains.

## 1 Introduction

According to the China Antimicrobial Resistance Surveillance System (http://www.carss.cn), *Staphylococcus aureus* has long been the most frequently isolated Gram-positive pathogen in clinical settings. Its diverse genetic background and arsenal of virulence factors allow it to cause a wide spectrum of infections, ranging from superficial skin lesions such as folliculitis to life-threatening systemic diseases, including osteomyelitis, septic arthritis, bacteremia, and pulmonary infections [1]. Restriction-modification (RM) systems constitute a major obstacle to molecular mechanistic studies on these clinical *S. aureus* isolates. They function by recognizing and degrading exogenous DNA that lacks the host-specific methylation pattern, leading to transformation failure and rendering conventional gene editing and advanced genetic manipulations unfeasible [2]. These systems are categorized into types I, II, III, and IV, and can exist in various combinations within different strains, all of which impede genetic manipulation to some degree. The co-occurrence of type I and type II systems is a particularly common and challenging configuration, often rendering standard transformation attempts unsuccessful and thereby stalling functional studies [3,4].

The current strategy to overcome these barriers integrates the "SyngenicDNA" approach (to evade type I RM systems) with "plasmid artificial modification (PAM)" (to evade type II RM systems) [[4], [5], [6]]. This strategy involves two key steps: first, introducing synonymous mutations into the plasmid backbone to eliminate type I restriction motifs; second, transforming the engineered plasmid into a specialized *E. coli* strain (e.g., EC135) that expresses the type II methyltransferase M.*Sau*3AI, thereby performing the requisite type II methylation. While effective, this strategy depends on access to specifically engineered bacterial strains. Moreover, if the newly introduced DNA contains host-specific methylation motifs, additional plasmid sequence adjustments may be required, which increases operational complexity and limits its broad applicability.

To address these limitations, we aimed to develop a more streamlined strategy: constructing a restriction-deficient, highly transformable intermediate host within the lineage of the target strain, thereby bypassing the initial transformation barrier. This strategy requires a two-stage process: first, analogous to the strategy of Monk et al. [7], we engineered an *E. coli* donor strain to express the host methyltransferases, mimicking the type I methylation pattern of the target *S. aureus* strain; second, we used these pre-methylated plasmids to knock out the genes encoding the type I and type II restriction endonucleases in the *S. aureus* chromosome. After this one-time construction, this intermediate host could serve as a universal shuttle, accepting plasmids from standard *E. coli* and producing them in a form readily transformable into the wild-type parental strain. To validate this concept, we selected the clinical isolate *S. aureus* XQ, a strain known to harbor both type I and II RM systems [3,4]. This strain was isolated from a fatal sepsis case and belongs to the globally disseminated sequence type 121 (ST121) [8]. Based on comprehensive sequencing data and in silico predictions from the REBASE database, we observe that methylation patterns are highly conserved among *S*. *aureus* isolates of the same ST. For example, all ST121 strains share identical type I recognition sites (GGA(N)_6_CCT and GAC(N)_6_TAYG) and the type II recognition site (GATC) with strain XQ [3,4]. This conservation suggests that an intermediate strain developed for XQ should be applicable across the entire ST121 lineage.

In this study, we successfully constructed a restriction-deficient intermediate host for the ST121 *S. aureus* isolate XQ. Building on this, we optimized the shuttle plasmid by reducing it to a single, flexibility selectable resistance marker. We assessed and ruled out the feasibility of a stable dual-plasmid system in this strain. Finally, we demonstrated the utility of this transformation system through conventional gene editing in XQ and its variants. This work provides a user-friendly and adaptable strategy to engineer tailored intermediate strains. We have demonstrated its ready applicability across multiple ST121 isolates, thereby establishing a validated framework for broader application in other *S. aureus* isolates harboring different combinations of type I and II RM systems.

## 2 Results

### 2.1 Construction and characterization of intermediate hosts

**Fig. 1:**
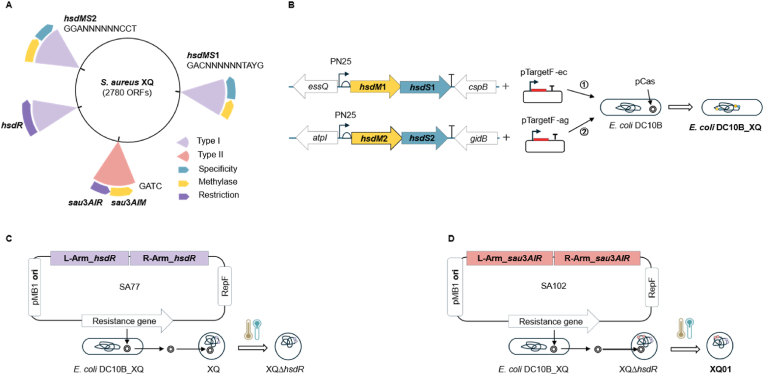
Construction of a restriction-deficient intermediate strain for *S. aureus* ST121 electrotransformation. (A) Schematic diagram of the RM systems in *S. aureus* XQ. The specific recognition sequences for the type I and type II RM systems are indicated. (B) Construction of the *E. coli* DC10B_XQ by integrating the *hsdMS1* and *hsdMS2* genes from XQ into the DC10B genome via CRISPR-Cas9. Two genomic loci, the intergenic regions *essQ*–*cspB* and *atpI*–*gidB*, were targeted for insertion. (C) Diagrammatic showing the strategy for deleting the type I restriction gene *hsdR* in *S. aureus* XQ via allelic exchange, yielding the intermediate mutant XQΔ*hsdR*. (D) Strategy for deleting the type II restriction gene *sau3AIR* in XQΔ*hsdR* via allelic exchange, generating the final restriction-deficient intermediate strain XQ01. Abbreviations: L-Arm_*xx*: left homologous arm of gene *xx*; R-Arm_*xx*: right homologous arm of gene *xx*. pTarget-ec: plasmid expressing a guide RNA targeting the *essQ*–*cspB* intergenic region. pTarget-ag: plasmid expressing a guide RNA targeting the *atpI*–*gidB* intergenic region. RepF: temperature-sensitive replicon for *S. aureus*. The plasmids corresponding to the profiles are designated SA77 and SA102.

**Fig. 2:**
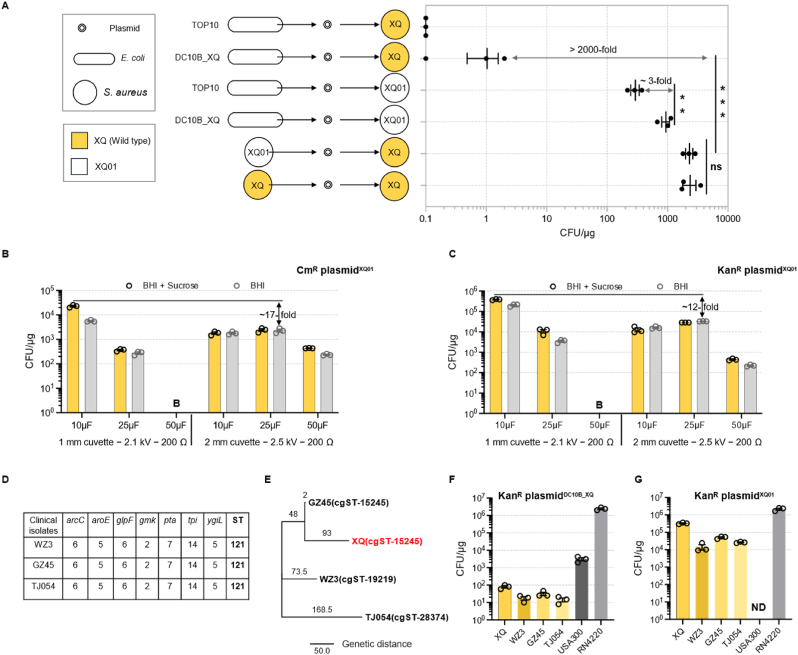
Optimization of transformation efficiency and evaluation of broad applicability within *S. aureus* ST121. (A) Transformation efficiency of *S. aureus* XQ and XQ01 with plasmids prepared from standard *E. coli* TOP10, engineered strain DC10B_XQ, and intermediate strain XQ01 under initial electroporation conditions (2.5 kV, 200 Ω, 25 μF, 2 mm cuvette gap; recovery in BHI broth). Values of zero are plotted as 0.1 for logarithmic visualization. Statistical analyses were performed using two-tailed lognormal t-tests. ∗∗∗P < 0.001, ∗∗P < 0.01; ns, not significant (P > 0.05). (B, C) Systematic optimization of electroporation conditions for transforming *S. aureus* XQ with plasmids derived from the intermediate strain XQ01. Fold change was calculated by comparing the optimal condition to the initial condition. (B) Plasmid carrying chloramphenicol resistance (Cmᴿ). (C) Plasmid carrying kanamycin resistance (Kanᴿ). Evaluated parameters included capacitance (10, 25, and 50 μF) and recovery medium composition (BHI with or without 0.5 M sucrose), under two cuvette/voltage conditions: 2.1 kV in a 1 mm cuvette and 2.5 kV in a 2 mm cuvette (both with 200 Ω resistance). Conditions marked with a bold ‘B’ resulted in a burst cuvette. (D) Multilocus sequence typing (MLST) confirms three clinical *S. aureus* isolates as ST121. The numbers represent the corresponding allelic profile for each of the seven housekeeping genes. (E) Genetic relatedness of XQ and the three other ST121 clinical isolates based on core genome multilocus sequence typing (cgMLST). The neighbor-joining phylogenetic tree was constructed from cgMLST data. The core genome sequence type (cgST) for each isolate is indicated in parentheses. Scale bar indicates genetic distance. (F, G) Electroporation efficiency of Kanᴿ plasmids from the engineered strains into various *S. aureus* strains. (F) Plasmid derived from DC10B_XQ. (G) Plasmid derived from XQ01. Electroporation was performed under the optimal conditions determined in panels B and C. Each symbol represents an independent biological replicate (*n* = 3). Data are presented as mean ± standard error of the mean (SEM). ND: not detected.

Analysis of the REBASE database and PacBio SMRT sequencing (BioProject ID: PRJNA1463998) confirmed that *S. aureus* XQ contains both type I and type II RM systems and lacks a type IV system (Fig. 1A and Supplementary Fig. S1). To overcome these restriction barriers, we first integrated the *hsdMS*1 and *hsdMS*2 genes from XQ into the *E. coli* DC10B genome via CRISPR-Cas9. Insertion sites were selected in the intergenic regions between *essQ* and *cspB,* and between *gidB* and *atpI,* as previously described [7]. The resulting strain was designated DC10B_XQ (Fig. 1B). Although plasmids isolated from DC10B_XQ could be electroporated into XQ, the transformation efficiency was remarkably low, approximately 1 colony-forming unit (CFU) per microgram of DNA under initial conditions (Fig. 2A). This result highlighted that the type II RM system also presents a major barrier to genetic transformation. To confirm this, we performed single knockouts of the type I (*hsdR*) and type II (*sau3AIR*) restriction genes in XQ using pre-methylated allelic exchange plasmids (pAEM_K derivatives). As expected, inactivation of s*au3AIR* (type II) improved electroporation efficiency far more markedly than inactivation of *hsdR* (type I) (Supplementary Fig. S2). We subsequently inactivated both genes to generate the electrocompetent intermediate strain XQ01 (Fig. 1C and D). Plasmids isolated from XQ01 exhibited over 2000-fold higher transformation efficiency in wild-type XQ than those from DC10B_XQ. There was no significant difference in transformation efficiency between plasmids prepared from XQ01 and those from wild-type XQ. Furthermore, plasmids prepared from standard *E. coli* strains (e.g., TOP10) could be efficiently transformed into XQ01. The transformation efficiency of plasmids from DC10B_XQ was three times higher than that of plasmids from TOP10 (Fig. 2A).

To further improve electroporation efficiency and assess the generality of the protocol, we systematically optimized key parameters. Using our initial standard conditions (2.5 kV, 200 Ω, 25 μF, 2 mm cuvette gap; recovery in BHI broth) and two different resistance-marker plasmids extracted from XQ01, we evaluated different cuvette gaps (1 or 2 mm), capacitance settings (10, 25, or 50 μF), and recovery media (BHI alone or BHI supplemented with sucrose). With resistance fixed at 200 Ω, both plasmids consistently showed that the optimal conditions for XQ were electroporation in a 1 mm cuvette at 2.1 kV with 10 μF capacitance, followed by recovery in BHI supplemented with 0.5 M sucrose (Fig. 2B and C). This optimized protocol increased the transformation efficiency to over 2 × 10^4^ CFU/μg, resulting in a >10-fold improvement over the starting conditions.

To validate the applicability of our engineered intermediate strains to other ST121 isolates, we selected three genetically distinct ST121 clinical strains (WZ3, GZ45, and TJ054) (Fig. 2D and E). Using the optimized electroporation protocol, we transformed each strain with plasmids isolated from both DC10B_XQ and XQ01. The restriction-deficient strain RN4220 and the well-characterized USA300 strain FPR3757 were included as controls (Fig. 2F and G). Since TJ054 is intrinsically resistant to chloramphenicol, a plasmid carrying a kanamycin resistance marker was used for all transformations to ensure consistency. The results demonstrated successful transformation of all four ST121 isolates (the three independent clinical isolates and XQ) by plasmids from both source strains. As anticipated, efficiencies differed markedly: plasmids from DC10B_XQ transformed these isolates at low efficiency (typically on the order of tens of CFU/μg), whereas plasmids from XQ01 achieved significantly higher efficiencies, exceeding 2 × 10^4^ CFU/μg. For the restriction-deficient control RN4220, transformation efficiencies were high (>10^6^ CFU/μg) with plasmids from both sources. In contrast, USA300 exhibited a different outcome: while the DC10B_XQ-derived plasmid was transformable, the XQ01-derived plasmid failed to yield transformants. This differential transformability aligns with the distinct RM system profiles documented for USA300 (types I and IV) and the donor strain XQ (types I and II) [4].

### 2.2 Streamlined shuttle plasmid design via cross-species promoter characterization

**Fig. 3:**
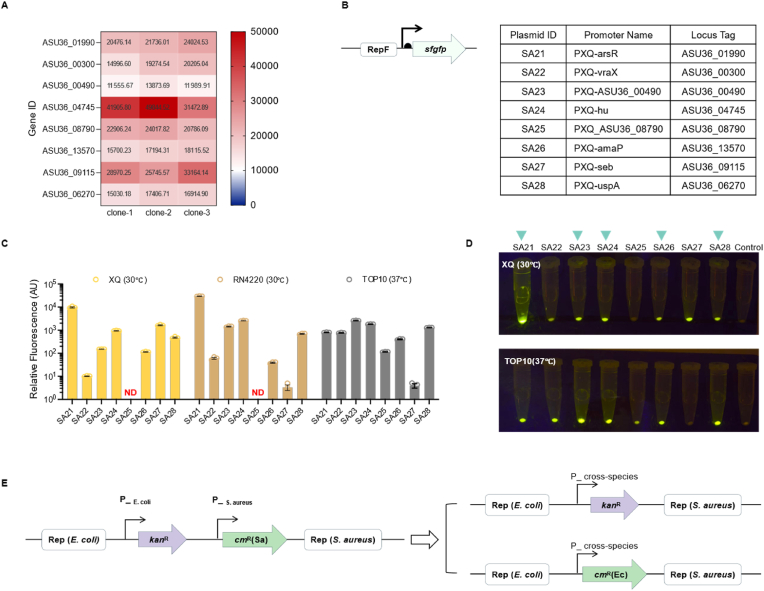
Characterization of cross-species promoters in *S. aureus* XQ and *E. coli* TOP10. (A) Heatmap of transcripts per million (TPM) values for eight highly expressed genes (TPM >10,000) selected from the transcriptome of *S. aureus* XQ. (B) Schematic of the eight *sfgfp* reporter plasmids containing putative promoters from the highly expressed genes in (A). (C) Comparative analysis of promoter activity by flow cytometry in *S. aureus* strains XQ and RN4220 and *E. coli* TOP10. Each symbol represents an independent biological replicate (*n* = 3). Data are presented as mean ± SEM. Fluorescence is presented in arbitrary units (AU). ND: not detected (signals below control levels). (D) Fluorescence of bacterial cell pellets expressing GFP under blue light excitation. Green triangles highlight promoters with relatively high activity in both XQ and TOP10. (E) Schematic of converting a dual-antibiotic-resistance shuttle plasmid into single-resistance plasmids using cross-species promoters. Abbreviations: Cmᴿ(Sa): chloramphenicol resistance gene for selection in *S. aureus* (e.g., from pCasSA); Cmᴿ(Ec): chloramphenicol resistance gene for selection in *E. coli* (e.g., from transposon Tn9); Rep(*E. coli)*: *E. coli*-specific replication origin; Rep(*S. aureus*): *S. aureus*-specific replication origin; P_*E. coli*: promoter active only in *E. coli*; P_*S. aureus*: promoter active only in *S. aureus*; P_cross-species: promoter active in both *E. coli* and *S. aureus*.

Early studies identified two classes of promoters in *S. aureus*. Class II promoters function exclusively in Gram-positive hosts, whereas Class I promoters also function in *E. coli* [9,10]. We characterized eight intergenic sequences (150–400 bp) from *S. aureus* XQ, located upstream of genes with high transcript-per-million (TPM) values as determined by transcriptome analysis (Fig. 3A). These sequences were used to construct eight sfGFP reporter plasmids (Fig. 3B). Flow cytometry revealed that five out of the eight promoters exhibited cross-species activity, functioning in both *E. coli* and *S. aureus* (Fig. 3C and D). Notably, the activity patterns of these five promoters were conserved between the two *S. aureus* strains tested, XQ and RN4220 (Fig. 3C).

Conventional shuttle plasmids typically contain two antibiotic-resistance markers, such as kanamycin resistance for selection in *E. coli* and chloramphenicol resistance for *S. aureus*. However, in cases where the target *S. aureus* strain exhibits inherent resistance to its designated selection marker, that marker needs to be replaced. To facilitate flexible antibiotic selection, we replaced the original promoter with a cross-species promoter and constructed plasmids carrying only a single resistance gene (Fig. 3E). Testing revealed that the cross-species promoter effectively drove the expression of antibiotic resistance genes in both *E. coli* and *S. aureus* when combined with either the *aph*(3′)-II (or *npt*II) gene (conferring kanamycin resistance) or the *cat* gene from transposon Tn9 (conferring chloramphenicol resistance; designated as Cmᴿ(Ec)). However, when the same cross-species promoter was used to express a *cat* gene derived from a conventional *E. coli*–*S. aureus* shuttle plasmid (e.g., pCasSA; designated Cmᴿ(Sa)) in *E. coli*, almost no transformants were obtained. The codon adaptation index (CAI) of Cmᴿ(Sa) in *E. coli* was only 0.56, below the generally accepted threshold of 0.7, implying that codon bias likely contributed to its poor expression [11,12]. Therefore, these results establish that a single cross-species promoter, when driving a compatible resistance gene, enables antibiotic selection in both hosts.

### 2.3 Stability evaluation of different replicons in *S. aureus* XQ

**Fig. 4:**
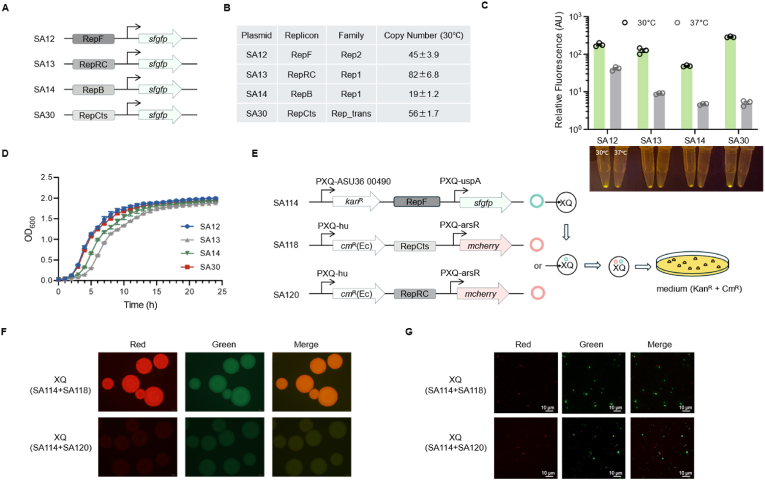
Characterization of plasmid replicons in *S. aureus* XQ. (A) Design of four sfGFP-expressing reporter plasmids, each bearing a distinct *S. aureus* replicon. (B) Replicon families and copy numbers of the sfGFP reporter plasmids in *S. aureus* XQ at 30 °C. Replicon families were identified using PlasmidFinder. Plasmid copy number per chromosome was determined by digital PCR using mid-log phase bacterial cells. (C) Fluorescence comparison of *S. aureus* strains containing the reporter plasmids with distinct replicons at 37 °C and 30 °C. Upper panel: Flow cytometry analysis. Lower panel: Fluorescence of cell pellets under blue light excitation. All cells were harvested at the late-log phase. Fluorescence data are presented in arbitrary units (AU). (D) Growth curves of XQ strains bearing the reporter plasmids with distinct replicons in BHI medium at 30 °C with continuous shaking. Data points represent mean ± SEM (*n* = 3 biological replicates). (E) Workflow for introducing two fluorescent reporter plasmids into *S. aureus* XQ via sequential electroporation to verify replicon compatibility. (F) Fluorescence of *S. aureus* XQ colonies co-harboring red and green fluorescent protein plasmids on dual-antibiotic agar plates. Colonies were imaged after growth. Note the reduced fluorescence intensity in colonies carrying the RepRC replicon (Rep1 family). (G) Single-cell fluorescence microscopy of bacteria harboring both fluorescent plasmids after growth to late-log phase in liquid medium with dual-antibiotics. Only a minority of individual cells co-expressed both fluorescent proteins. SA12, SA13, SA14, SA30, SA114, SA118, and SA120 are plasmid names.

Dual-plasmid systems can facilitate modular design and independent regulation. To evaluate replicon coexistence stability in *S. aureus* XQ, we constructed four reporter plasmids, each harboring a distinct, commonly used replicon: RepF (a temperature-sensitive variant of pE194), RepCts (a temperature-sensitive derivative of pT181), RepB (from pUB110), and RepRC (from pRAB11) (Fig. 4A). According to the online tool PlasmidFinder, these replicons belong to three distinct families: RepRC and RepB belong to the Rep1 family, RepF to the Rep2 family, and RepCts to the Rep_trans family [13,14]. The copy numbers of the four replicons at 30°C were determined by digital PCR (Fig. 4B). Interestingly, the RepRC replicon, which had the highest copy number, did not yield the highest reporter expression as measured by flow cytometry (Fig. 4C). Moreover, strains carrying either RepRC or the lower-copy-number RepB (from the same replicon family) exhibited markedly slower growth at 30°C compared to strains harboring the other two replicons (Fig. 4D), suggesting a strong metabolic burden imposed by these two replicons in *S. aureus* XQ. All four replicons also exhibited higher expression levels at 30 °C than at 37 °C (Fig. 4C). Therefore, we assessed whether these replicons from different families could stably coexist at 30 °C. We sequentially introduced simplified shuttle reporter plasmids into *S. aureus* XQ: first a green fluorescent plasmid carrying the RepF replicon (Kan^R^), then a red fluorescent plasmid harboring a replicon from a different family (Cmᴿ(Sa)) (Fig. 4E). Single colonies selected on solid medium containing dual antibiotics exhibited fluorescence from both reporter proteins (Fig. 4F). However, after culturing these colonies in liquid medium with both antibiotics, fluorescence microscopy revealed that the proportion of cells expressing both fluorescent proteins was very low (Fig. 4G). Moreover, subculturing the dual-resistant cells in liquid medium containing both antibiotics failed to maintain growth; growth could only be re-established by streaking onto solid dual-selection plates (data not shown). Although replicons from different families can be used to construct stable dual-plasmid systems in some *S. aureus* strains [15,16], in the clinical isolate XQ, the tested replicons could not stably coexist under the evaluated conditions and are therefore unsuitable for a dual-plasmid system in this strain.

### 2.4 Efficient gene editing of *S. aureus* XQ using simplified allelic exchange plasmids

**Fig. 5:**
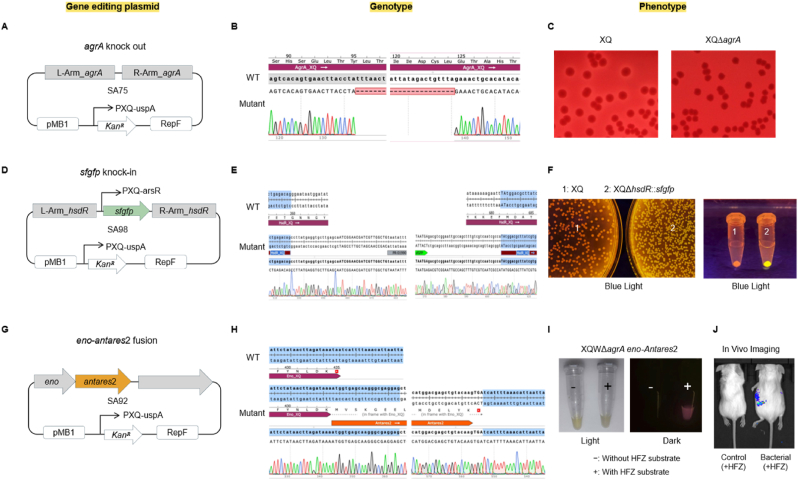
Construction and phenotypic validation of gene mutants in *S. aureus* XQ and its derivatives. (A) Schematic of the allelic exchange plasmid for deleting the *agrA* in XQ. (B) Sequencing confirming the *agrA* deletion in *S. aureus* XQ. (C) Comparison of hemolytic phenotypes on sheep blood agar between the *agrA* knockout mutant and the wild-type XQ. (D) Schematic of the allelic exchange plasmid for inserting the *sfgfp* gene into the *hsdR* locus of *S. aureus* XQ. (E) Sequencing confirming the *sfgfp* knock-in at the *hsdR* locus in XQ. (F) Comparison of fluorescence between the *sfgfp* knock-in mutant and the wild-type XQ cultured on solid agar plates and in liquid media, visualized under blue light. (G) Schematic of the allelic exchange plasmid for fusing the *antares2* luciferase gene downstream of the chromosomal *eno* gene in the *S. aureus* variant XQW (a non-pigmented derivative of XQ). (H) Sequencing confirming the *eno-antares2* fusion in XQW. (I) Bioluminescence produced by the XQWΔ*agrA eno-antares2* reporter strain was compared between conditions with and without the substrate hydrofurimazine (HFZ), under both light and dark exposure. (J) In vivo bioluminescence imaging of BALB/c mice. The right mouse (bacterial) was injected with 1 × 10^5^ CFU of the XQWΔ*agrA eno-antares2* reporter strain via the tail vein, followed by intraperitoneal injection of 1 μmol HFZ 1 h later. The left mouse (control) received HFZ only. Imaging was performed using a small animal imaging system. SA75, SA92, and SA98 are plasmid names. Abbreviations: *antares2*: gene for luciferase derivative; *eno*: gene for enolase; L-Arm_XX: left homologous arm of gene XX; R-Arm_XX: right homologous arm of gene XX.

The allelic exchange method using temperature-sensitive shuttle plasmids is a widely adopted gene-editing approach in *S. aureus*. We constructed a simplified allelic exchange backbone (pAEM_K) carrying a kanamycin resistance marker. The *agrA* gene regulates the expression of δ- and α-hemolysin, and its deletion leads to a non-hemolytic phenotype [17]. Using this backbone, we designed a derivative plasmid containing homology arms flanking the *agrA* gene and introduced it into XQ through the intermediate strain XQ01 (Fig. 5A). After culturing and dilution, wild-type XQ and sequence-validated mutants were plated on blood agar. Clear hemolytic halos were observed around wild-type colonies but not around the *agrA* knockout strains (Fig. 5B and C).

Genomically integrated reporter genes are valuable for tracking bacterial distribution. To this end, we constructed two additional reporter strains. For fluorescence reporting, we designed a plasmid to insert *sfgfp* into the *hsdR* locus (Fig. 5D). Sequence-confirmed mutants (Fig. 5E) exhibited strong green fluorescence under blue light excitation on both agar plates and in liquid culture (Fig. 5F). For bioluminescence reporting, we adopted the strategy of Shang et al. [18], constructing a plasmid to fuse the *antares2* gene to the 3′ end of the chromosomal *eno* gene in the *S. aureus* variant XQWΔ*agrA* (Fig. 5G). We selected this genetic background because the XQW strain (a non-pigmented variant of XQ) promotes biofilm formation and membrane vesicle production [19,20], and the *agrA* deletion contributes to attenuation [17,21], making it a suitable platform for developing attenuated therapeutic vectors. Mutants confirmed by sequencing emitted intense orange-red bioluminescence upon addition of the substrate hydrofurimazine (HFZ), visible to the naked eye in the dark (Fig. 5H and I). Furthermore, bioluminescence from mice injected with these engineered strains and HFZ was successfully detected using a small-animal in vivo imaging system (Fig. 5J). These results demonstrate that our streamlined plasmid system can successfully generate functional mutants in the XQ strain lineage.

## 3 Conclusion

Clinical isolate *S. aureus* ST121, such as strain XQ, carries both type I and type II RM systems, which severely hinder plasmid transformation [12]. To develop a generalizable genetic toolkit for such dual-RM strains, we established a two-step electroporation strategy. First, we engineered an *E. coli* strain to express the type I methyltransferase from *S. aureus* XQ, enabling the pre-methylation of shuttle plasmids and their initial, albeit low-efficiency, delivery into XQ. Second, using these modified plasmids, we sequentially knocked out the genes encoding the type I and type II restriction endonucleases in XQ. This created a restriction-deficient intermediate strain, which elevated the transformation efficiency to >10^4^ CFU/μg of DNA. Furthermore, plasmids isolated from this intermediate strain were successfully electroporated into multiple other ST121 clinical isolates that share the identical methylation pattern, confirming the broad applicability of the strategy. Additionally, to streamline shuttle plasmid design, we identified and employed a cross-species promoter, which facilitated flexible antibiotic marker exchange. Using these optimized plasmids, we successfully performed various genetic manipulations in XQ, including gene knockout, knock-in, and reporter fusions. This proof-of-concept work provides a novel tool for overcoming electroporation barriers in other clinical *S. aureus* isolates harboring distinct type I and type II RM systems.

## 4 Discussion

In this study, we employed a two-step strategy to construct engineered strains *E. coli* DC10B_XQ and *S. aureus* XQ01, enabling high-efficiency electroporation. While the construction of XQ01 proceeded smoothly, the engineering of the *E. coli* donor strain, despite a clear design, presented significant obstacles. From these challenges, we have identified several avoidable complications. Specifically, during genome integration using the CRISPR-Cas9 system, we encountered two major issues. The first hurdle was obtaining the donor DNA fragment. Since we were unsuccessful to construct a plasmid for overexpressing *S. aureus hsdMS* in *E. coli,* we ultimately adopted an unconventional approach to amplify the required fragment (detailed in Materials and Methods). Commercial synthesis of this fragment could circumvent the operational complexity associated with this step. The second difficulty arose during the sequential integration of the two *hsdMS* modules. After successfully integrating the first module, the efficiency for integrating the second module decreased markedly. For the first integration, screening 24 colonies yielded a correct clone. For the second integration, screening approximately 300 single colonies identified only two clones with PCR products of the expected size. Sequencing revealed that one clone (Clone 1#, i.e., DC10B_XQ) harbored a fully correct integration, while the other (Clone 3#) contained a premature stop codon within the *hsdM*2 gene of the *hsdMS*2 module (Supplementary Fig. S3A). Given the high conservation of *hsdM* across strains [22] and the prior successful use of a single *hsdM* by Monk et al. [7], we compared the plasmid modification efficiency from Clone 1# and Clone 3# by electroporating their derived plasmids into *S. aureus* XQ. Both plasmids were successfully electroporated, with the efficiency from Clone 1# being approximately twice that of Clone 3# (Supplementary Fig. S3B). Since subsequent gene editing relies on electroporating allelic exchange plasmids into *S. aureus*, either clone could be used for downstream applications. Based on these observations, we speculate that employing the CRISPR-Cas system to integrate a fragment expressing only *hsdS2* in the second step might simplify the identification of positive clones.

The transformation efficiency achieved for the ST121 strain (>10^4^ CFU/μg) marks a substantial improvement, enabling genetic manipulation in a previously non-transformable clinical isolate. This efficiency, however, remains lower than the >10^6^ CFU/μg reported for some other *S. aureus* strains [4,6]. The observed discrepancy is unlikely to originate from our engineered intermediate strain, XQ01, as plasmids prepared from it showed no significant difference in transformation efficiency compared to those modified by the wild-type XQ strain when electroporated into the native host (Fig. 2A). Therefore, further optimization to bridge this gap may focus on other factors, such as plasmid topology, growth media, reagents for competent cell preparation, and operational procedures [[23], [24], [25]]. For example, we observed that freshly extracted plasmids exhibited a higher degree of supercoiling compared to those stored at 4 °C or −20 °C. We refer to these as highly supercoiled and moderately supercoiled plasmids, respectively. The transformation efficiency of the highly supercoiled plasmids was approximately threefold higher than that of the moderately supercoiled plasmids (Supplementary Fig. S4)

In *S. aureus*, gene editing via allelic exchange commonly employs temperature-sensitive replicons like RepF. During our work, we noted that cultivating bacteria at 42 °C under antibiotic selection to drive plasmid integration carried a risk of introducing single nucleotide polymorphisms (SNPs) into the genome. The bacterial genome, however, remained stable in the following steps conducted at 30°C and 37 °C. Consequently, to ensure absolute sequence fidelity for studies where precise genotype is critical, whole-genome re-sequencing of edited clones is advisable. Alternatively, adopting a temperature-sensitive replicon with a lower permissive temperature, such as the lactic acid bacteria-derived replicon in the pIMAY-Z plasmid, may further minimize this risk by ceasing plasmid replication at 37°C [7,26].

*S. aureus* harbors four major RM systems that restrict foreign DNA entry [27]. In an analysis of 1980 *S. aureus* strains from the REBASE database by Yang et al. [4], 242 possessed a type IV system, 1380 had a type I system, 7 contained both type I and type IV systems along with a type III system, and 138 carried a type II system. The type IV RM system is the most readily circumvented, as plasmids can be propagated in *dcm*-deficient *E. coli*, such as DC10B, to avoid restriction [28]. For strains harboring only type I, or both type I and type IV systems, transformation can be achieved by integrating the cognate type I *hsdMS* genes of *S. aureus* into DC10B to mimic the host-specific methylation pattern [7], or via the “SyngenicDNA” approach [6]. For *S. aureus* strains carrying type I, III, and IV systems simultaneously, Corvaglia et al. [29] identified a type III-like restriction endonuclease. After eliminating the type I and type IV restrictions, inactivation of this type III-like enzyme resulted in markedly improved electroporation efficiency. In this subset, type II RM systems in *S. aureus* exist alone or co-occur with type I or type IV systems, but not with type III. To overcome type II restriction, strategies include the plasmid artificial modification (PAM) approach [5] and, as demonstrated here, knockout of the host restriction enzyme. We constructed a strain (XQ Δ*hsdR*) that simulates the presence of only a type II RM system. Plasmids propagated in *E. coli* could be electroporated into this strain at low efficiency. Subsequent knockout of the type II restriction endonuclease *Sau*3AI enabled high-efficiency electroporation (Supplementary Fig. S2). Collectively, our work expands the repertoire of electroporation strategies for *S. aureus*, suggesting that theoretically any clinical *S. aureus* isolate can be rendered highly transformable.

## 5 Materials and methods

### 5.1 Bacteria, plasmids and reagents

Bacterial strains utilized in this study are summarized in Supplementary Table S1. Relevant plasmids are detailed in Table S2. The main promoters used in this study, and the sequences of Cmᴿ(Ec) and Cmᴿ(Sa), are listed in Table S3.

Unless otherwise stated, *S. aureus* strains were cultured in Brain Heart Infusion (BHI) broth, and *E. coli* strains were grown in Luria–Bertani (LB) medium. Plasmid mini-prep and midi-prep for *E. coli* were performed using UE-MN-P and UE-MD-P kits from UElandy. For *S. aureus,* plasmid mini-prep and midi-prep were carried out with the Wizard® Plus SV Minipreps DNA Purification System and PureYield™ Plasmid Midiprep System (Promega), respectively. PCR products were isolated using the Universal DNA Purification Kit (TIANGEN). PCR reagents: PrimeSTAR Max DNA Polymerase was purchased from TAKARA, KOD FX DNA polymerase from TOYOBO, Platinum SuperFi II PCR Master Mix from ThermoFisher, and Taq Master Mix from Vazyme. Reagents for Gibson assembly were purchased from ABclonal, while enzymes for Golden Gate assembly were obtained from New England Biolabs (NEB). L-(+)-Arabinose was purchased from Sigma and was used at 0.2% (w/v) in *E. coli*. The antibiotics and their final concentrations used in this study were as follows: spectinomycin (Spe^R^, 100 μg/mL), chloramphenicol (Cm^R^, 20 μg/mL for *E. coli*, 10 μg/mL for *S. aureus)*, and kanamycin (Kan^R^, 50 μg/mL for *E. coli*, 100 μg/mL for *S. aureus*). Sanger sequencing was performed by Sangon Biotech or Tsingke Biotechnology. PacBio SMRT sequencing, methylation motif analysis, and mutant resequencing were conducted by Haorui Genomics.

### 5.2 Construction of DC10B_XQ using CRISPR-Cas9

The *E. coli* strain DC10B_XQ was constructed using the CRISPR-Cas9 system described by Jiang et al. [30]. Two gRNA plasmids were made: pTargetF-ec (spacer: 5′-aatatcagtctgctaaaaat-3′) for the *essQ*-*cspB* (ec) site, and pTargetF-ag (spacer: 5′-ggcaaaacaaagtgcgtaag-3′) for the *atpI*-*gidB* (ag) site. Both were assembled by inserting the spacer into pTargetF (Addgene #62226) via Golden Gate assembly and confirmed by sequencing.

The donor DNA for each locus was constructed in separate steps. First, a "PN25-RBS-T7TE" fragment was synthesized by long-primer PCR (sequence provided in Supplementary Table S4). For integration at the ec-site: the left homology arm (HHL-ec, ∼500 bp) was amplified from the *E. coli* DC10B genome using primers cspB_F/R, and the right homology arm (HHR-ec, ∼500 bp) was amplified from the *E. coli* DC10B genome using primers essQ_F/R. The PN25-RBS-T7TE fragment, together with HHL-ec and HHR-ec, was Gibson-assembled into a pMB1 backbone to create a plasmid containing the ec-site homology arms. Subsequently, this plasmid was amplified with primers T7TE_F and RBS_R and assembled with the *hsdMS1* coding sequence (amplified from the *S. aureus* XQ genome using primers hsdMS1_F/R) to generate the ec-site pre-plasmid (without transformation). Then, using 1 μL of this pre-plasmid as template, an Inverse PCR was performed with primers Inverse_F/R designed against the *hsdM1* gene. The final linear donor for the ec-site was PCR-amplified from this product (diluted 100–1000 fold) using primers cspB_F and essQ_R. Using the ec-site donor and pTargetF-ec, *hsdMS1* was first integrated into the DC10B genome via CRISPR-Cas9, yielding a strain designated DC10B_XQ-hsdMS1, which was confirmed by sequencing.

Next, the ag-site donor was obtained using the same method. The left homology arm (HHL-ag, ∼500 bp) was amplified from the DC10B genome using primers atpI_F/R, and the right homology arm (HHR-ag, ∼500 bp) was amplified from the DC10B genome using primers gidB_F/R. The PN25-RBS-T7TE fragment, together with HHL-ag and HHR-ag, was Gibson-assembled into a pMB1 backbone to create a plasmid containing the ag-site homology arms. Subsequently, this plasmid was amplified with primers T7TE_F and RBS_R and assembled with the *hsdMS2* coding sequence (amplified from the *S. aureus* XQ genome using primers hsdMS2_F/R) to generate the ag-site pre-plasmid (without transformation). Using 1 μL of this pre-plasmid as template, an Inverse PCR was performed with the same primers Inverse_F/R designed against the *hsdM2* gene. The final linear donor for the ag-site was PCR-amplified using primers gidB_F and atpI_R. Finally, using the ag-site donor and pTargetF-ag, *hsdMS2* was integrated into the DC10B_XQ-*hsdMS1* strain. The resulting final strain, DC10B_XQ, was verified by Sanger sequencing to confirm the correct insertion of both *hsdMS1* and *hsdMS2*. All primers are listed in Supplementary Table S5, and the donor DNA sequences are provided in Supplementary Table S6.

### 5.3 Construction of *S. aureus* allelic exchange plasmids

All *S. aureus* allelic exchange plasmids were constructed using the backbone plasmid pAEM_K (GenBank: PZ351570) and assembled via Gibson assembly. The backbone plasmid pAEM_K was constructed via Gibson assembly and comprises three core elements: (i) a temperature-sensitive origin of replication (RepF) for maintenance in *S. aureus*; (ii) a pMB1-derived origin of replication for propagation in *E. coli*; and (iii) the cross-species constitutive promoter PXQ-ASU36_00490, driving expression of the *aph(3′)-II* gene which confers kanamycin resistance in both *E. coli* and *S. aureus*. Key features of this plasmid are summarized in Supplementary Table S2. For each construct, approximately 1 kb homology arms were amplified from *S. aureus* XQ genomic DNA. The key primers used are listed in Supplementary Table S5, and the complete sequences of all donor DNA fragments are provided in Supplementary Table S6. For construction of the *hsdR*:*sfGFP* allele, homology arms flanking the insertion site were amplified using primers ΔhsdR_up_F/R and ΔhsdR_dn_F/R, and the sfGFP coding sequence was amplified using primers sfGFP_F/R. For the *agrA* knockout construct, the upstream and downstream homology arms were amplified using primer pairs ΔagrA_up_F/R and ΔagrA_dn_F/R, respectively. Similarly, the *sau3AIR* knockout construct was generated using homology arms amplified with primers Δsau3AIR_up_F/R and Δsau3AIR_dn_F/R. For the C-terminal *eno*:*antares2* fusion, the downstream homology arm was amplified using primers eno_antares2_arm_F/R, and the *antares2* coding sequence was amplified with primers antares2_F/R. In each case, the respective PCR fragments were assembled with the linearized pAEM_K backbone via Gibson assembly. All final plasmid constructs were confirmed by Sanger sequencing.

### 5.4 Construction of *S. aureus* reporter plasmids

To investigate the impact of different replicons on reporter gene expression under identical conditions, we constructed a series of isogenic reporter backbones that differ only in their *S. aureus* replication origin. Four distinct replicons were employed: RepF, RepCts, RepB, and RepRC. RepF is the commonly used temperature-sensitive variant of the pE194 replicon. RepRC is derived from the pRAB11 plasmid. RepB is derived from the pUB110 plasmid. The RepCts replicon was commercially synthesized based on the sequence of plasmid pCN-EF2132tet (Addgene ID: 107191), which carries a temperature-sensitive derivative of the *S. aureus* replicon pT181 (pT181cop-634ts). All fragments were assembled into an otherwise identical shuttle vector backbone using Gibson assembly, yielding the final reporter plasmids SA12, SA13, SA14, and SA30. The co*mplete sequ*ences of these four plasmids have been submitted to GenBank (accession numbers:PZ366119-PZ366122).

### 5.5 Plasmid extraction from *S. aureus* (Midiprep)

Plasmids were extracted from *S. aureus* strains using the PureYield™ Plasmid Midiprep System (Promega). The procedure generally followed the manufacturer's protocol, with a key modification to improve cell wall lysis for this Gram-positive bacterium. Briefly, 50 mL of an overnight bacterial culture was pelleted by centrifugation and resuspended in 3 mL of Cell Resuspension Solution. Lysostaphin was added to a final concentration of 100 μg/mL, and the suspension was incubated at 37 °C for 1 h to facilitate peptidoglycan digestion. The subsequent steps, including the addition of Endotoxin Removal Wash Solution, were carried out using a vacuum manifold as recommended in the kit instructions. Purified plasmids were finally eluted in 400 μL of ultrapure water using the Eluator™ Vacuum Elution Device.

### 5.6 Preparation of electrocompetent *S. aureus* cells

*S. aureus* strains were reactivated through streaking on a BHI plate. Then a single colony was inoculated into BHI broth and cultured at 37 °C (or 30 °C if plasmids were present). The saturated culture was diluted 1:100 in 100 mL fresh BHI broth with shaking about 2 h until OD_600_ reached middle-log phase (OD_600_ ≈ 1.5). The cells were chilled on ice for 30 min and then harvested by centrifugation at 4 °C, 6000 rpm for 15 min. Cells were washed four times by centrifugation. After each wash, the supernatant was discarded. The first wash used an equal volume of sterile ice-cold 0.5 M sucrose solution, followed by washes with 1/2, 1/4, and 1/8 vol of the solution. After the last wash, the cells were resuspended in 0.5 M sucrose, aliquoted into pre-chilled sterile centrifuge tubes in volumes of 100 μL per portion and stored at −80 °C for long-term preservation.

### 5.7 Electrotransformation protocol for efficiency comparison

All electroporation experiments were performed using 1 μg of plasmid DNA and 100 μL of electrocompetent cells to ensure consistency. The first objective was to compare the transformation efficiency of plasmids harboring different methylation patterns into both wild-type and intermediate *S. aureus* strains. For this comparison, electroporation was carried out under the conditions routinely used in our laboratory (2 mm cuvette at 2.5 kV, 200 Ω, and 25 μF), followed by recovery in BHI medium. Subsequently, to identify the optimal conditions for transforming plasmids (extracted from the intermediate strain) into the wild-type *S. aureus* background, an extensive optimization was conducted. This included testing different cuvette gaps and capacitance settings (1 mm cuvette at 2.1 kV, 200 Ω with 10, 25, or 50 μF; and 2 mm cuvette at 2.5 kV, 200 Ω with 10, 25, or 50 μF) as well as comparing recovery media (BHI alone versus BHI supplemented with 0.5 M sucrose). Following electroporation under any condition, cells were recovered at 30 °C for 1 h, plated on selective agar, and incubated at 37 °C for 24 h prior to colony counting. Transformation efficiency was calculated and reported as CFU per microgram of plasmid DNA.

### 5.8 Transformation efficiency calculation

Transformation efficiency was determined by electroporating 1 μg of various plasmids into a panel of *S. aureus* strains. After plating on antibiotic-containing agar, colonies were counted. The transformation efficiency (CFU/μg) was calculated using the following formula: (Number of colonies on selective plate × Dilution factor)/(Mass of supercoiled DNA transformed (μg)). Data are presented as the mean ± SEM from n = 3 independent biological replicates.

### 5.9 Allelic exchange procedure

The allelic exchange procedure was adapted from Austin et al. [31]. The detailed protocol, with modifications, is described as follows:

Strain preparation: The allelic exchange plasmid was electroporated into the *S. aureus* strains of interest. Transformants were selected on BHI agar containing the appropriate antibiotics and incubated at 30 °C to obtain single colonies.

Day 1: A single colony harboring the allelic exchange plasmid was inoculated into 2 mL of BHI broth containing the appropriate antibiotics and incubated overnight at 42 °C (the nonpermissive temperature) with shaking.

Day 2: In the morning, 50 μL of the overnight culture was streaked onto a BHI agar plate containing antibiotics and incubated at 42 °C. In the evening, a single isolated colony was picked and inoculated into BHI broth (without antibiotics) for overnight incubation at 30 °C (the permissive temperature) with shaking.

Day 3: The culture from Day 2 evening was streaked onto a BHI agar plate (without antibiotics) and incubated at 30 °C.

Day 4: In the morning, single colonies were picked and patched onto fresh BHI agar plates (without antibiotics) for incubation at 30 °C. Colony PCR was performed simultaneously using primers flanking the homology arms to screen for the desired mutation. Note that the initial denaturation time is ideally ≥5 min, and KOD FX polymerase is preferred. If Taq polymerase is used for colony PCR, it is advisable to keep the concentration of *S. aureus* cells as low as possible. In the evening, clones yielding PCR products of the expected size were inoculated into 2 mL of antibiotic-free BHI broth and incubated overnight at 37 °C with shaking. These PCR products were subsequently subjected to Sanger sequencing for verification.

Day 5: In the morning, the overnight cultures confirmed by sequencing were diluted 1:20,000 into 10 mL of fresh, antibiotic-free BHI broth. In the evening, these cultures were again diluted 1:20,000 in antibiotic-free BHI broth and incubated overnight at 37 °C with shaking.

Day 6: In the morning, the cultures were streaked onto BHI agar plates (without antibiotics) and incubated at 37 °C. In the evening, single colonies were patched onto two parallel plates: BHI agar without antibiotics and BHI agar containing the appropriate antibiotics. Clones that grew only on the antibiotic-free plate (i.e., antibiotic-sensitive) were selected as candidate mutants that had lost the plasmid. Finally, verification was performed by colony PCR or next-generation sequencing (NGS), as needed.

### 5.10 Flow cytometric analysis of fluorescence

For fluorescence measurement, bacterial cells were prepared as follows: three single colonies from freshly streaked plates were inoculated into 1 mL of medium supplemented with the appropriate antibiotics in a 96-deep-well plate (2.2 mL well volume) and cultured to saturation using a microplate thermo shaker with shaking at 1000 rpm. The saturated cultures were then diluted 1:1000 into fresh medium containing the appropriate antibiotics in a new 96-deep-well plate (1 mL per well). Specifically, *E. coli* strains were grown in LB medium at 37 °C, and *S. aureus* strains were grown in BHI medium at 30 °C. After 24 h of cultivation, cells were harvested.

Relative fluorescence intensity of bacterial cells was measured using the FongCyte flow cytometer (Challenbio, China) with the following settings: FSC 1000, SSC 400, FITC 480. Before analysis, cells were diluted to a density of 10^6^–10^7^ CFU/mL in sterile PBS. At least 20,000 events were collected per sample and analyzed by FlowJo (version 10.4, Treestar, USA). The median fluorescence of each sample was calculated after subtracting the autofluorescence of *S. aureus* cells. All data were plotted with GraphPad Prism (version 7.0, La Jolla, USA).

### 5.11 Quantification of plasmid copy number by digital PCR (dPCR)

The intracellular plasmid copy number in *S. aureus* XQ was quantified using digital droplet PCR (ddPCR). Briefly, strains harboring green fluorescent reporter plasmids with different replication origins were cultured to the mid-log phase at 30 °C, and bacterial cells were collected by centrifugation (6000 rpm, 15 min), heat-inactivated at 65 °C for 10 min, and then shipped on dry ice to a service provider (Sangon Biotech) for analysis. Following total DNA extraction, ddPCR amplification and copy-number quantification were performed by the provider using a Bio-Rad QX200 system, with the plasmid-encoded *sfgfp* gene as the target and the single-copy chromosomal *nuc* gene as the genomic reference. Primer and probe sequences for *sfgfp* (sfgfp-F,sfgfp-R,sfgfp-P) and *nuc* (nuc-F,nuc-R,nuc-P) are listed in Supplementary Table S5. The plasmid copy number per cell was calculated as the ratio of the plasmid (*sfgfp*) copy concentration to the chromosomal (*nuc*) copy concentration, with three technical replicates performed per sample.

### 5.12 Growth curve assay

For each *S. aureus* strain, three independent single colonies were picked from a freshly streaked plate. Each colony was inoculated separately into BHI broth (containing the corresponding antibiotic) and incubated at 30 °C until saturation. Each of these three independent cultures was then diluted 1:100 into fresh BHI broth (with antibiotic) and dispensed into separate wells of a sterile 96-well microplate (200 μL per well). Blank control wells were included. The plate was placed in a Tecan microplate reader and incubated at 30 °C with continuous shaking. The OD_600_ was measured every hour until the values plateaued. Growth curves were plotted with incubation time as the x-axis and the averaged OD_600_ (blank-subtracted) from the three biological replicates as the y-axis.

### 5.13 *Sau*3AI genomic DNA digestion assay

To assess the functional activity of the type II restriction-modification system, genomic DNA from four representative ST121 isolates (XQ, WZ3, GZ45, TJ054) and the control strain RN4220 was subjected to digestion with *Sau*3AI (NEB). For each reaction, 1 μg of genomic DNA was incubated with 5 units of *Sau*3AI in 1× NEBuffer r1.1 at 37 °C for 1 h. A no-enzyme control (DNA incubated in buffer only) was included for each strain. After digestion, samples (equivalent to 100 ng of input DNA) alongside undigested controls and the GeneRuler DNA Ladder (Thermo Scientific) were separated on a 1% agarose gel in 1× TAE buffer. The gel was stained with SYBR Safe DNA Gel Stain (Invitrogen) and visualized under UV light. Resistance to digestion was defined by the persistence of a high-molecular-weight band comigrating with the undigested genomic DNA control.

### 5.14 Transcriptomic data analysis

Transcriptomic data were generously provided by a collaborator and were derived from *S. aureus* strain XQ. Three independent biological replicates were prepared; for each replicate, a single bacterial colony was picked to inoculate 3 mL of BHI broth in a 14 mL glass culture tube, followed by overnight incubation at 37 °C. Each pre-culture was then diluted 1:100 into fresh BHI medium in the same type of tube. After 6 h of growth at 37 °C, corresponding to the late-log phase (OD_600_ ≈ 5.3), cells were harvested. Total RNA was isolated using the E.Z.N.A. Bacterial RNA Kit (Omega Bio-tek), with a modified lysis step involving lysostaphin to enhance cell wall disruption. RNA sequencing libraries were constructed and sequenced on an Illumina platform by Sangon Biotech.

For bioinformatic analysis, raw sequencing reads were quality-trimmed using Trimmomatic v0.36. Clean reads were aligned to the reference genome of *S. aureus* XQ (GenBank assembly: GCA_001444345.1) using Bowtie2 v2.3.2, and gene expression levels were quantified as raw counts using featureCounts v1.6.0. Differential expression analysis was performed with DESeq2 v1.12.4 in R, with genes meeting the threshold of qValue ≤0.05 and |log_2_(fold change)| ≥ 1 considered significantly differentially expressed.

### 5.15 Hemolytic activity assay

The *agrA* knockout *S. aureus* XQ strains and the wild-type strain were separately cultivated at 37 °C until reaching saturation. Then, 1 μL of the bacterial culture was diluted 1:10^6^ in fresh BHI medium. Next, 100 μL of the bacterial suspension was plated on 5% (v/v) sheep blood agar plates and incubated at 37 °C for 24 h. After incubation, the plates were photographed to document hemolytic activity.

### 5.16 Small animal in vivo imaging experiment

Inoculum preparation: The inoculum was prepared from a late-log culture of *S. aureus* XQWΔ*agrA eno*-*antares2* grown in BHI broth to the late-log phase (∼6 h, from a stationary-phase culture diluted 1:100). At this point, the culture density was ∼1 × 10^9^ CFU/mL, as estimated from OD_600_. One milliliter of the culture was centrifuged, washed with sterile PBS, and resuspended in 1 mL of PBS. This suspension was then subjected to a 1:10 serial dilution in PBS three times to achieve a final concentration of ∼1 × 10^6^ CFU/mL.

Imaging: A female 6- to 8-week-old BALB/c mouse was injected via the tail vein with 1 × 10^5^ CFU of the XQWΔ*agrA eno*-*antares2* reporter strain. One hour later, 1 μmol of the substrate hydrofurimazine (HFZ) was injected into the peritoneal cavity. A wild-type mouse receiving only the substrate served as the negative control. Three minutes after substrate injection, bioluminescence signals from the kidneys were detected on the dorsal side of the mouse using an IVIS Lumina LT imaging system. The preparation of the HFZ substrate and the image acquisition parameters were based on a previous study [18].

## Ethical approval

Ethical approval Female BALB/c mice (6–8 weeks old, 16–20 g) were purchased from the Laboratory Animal Center of Third Military Medical University (Army Medical University). All animal experiments were approved by Laboratory Animal Welfare and Ethics Committee Of the Army Medical University (Approval No. AMUWE20223050, approved on 2022-2-28).

## Funding

This study was supported by the 10.13039/501100001809National Natural Science Foundation of China [82272341], and Chongqing Talent Program [CQYC20220303710].

## CRediT authorship contribution statement

**Yang Zheng:** Data curation, Formal analysis, Investigation, Methodology, Writing – original draft. **Chao Li:** Data curation, Investigation, Supervision, Writing – original draft. **Xinqi Huang:** Investigation, Validation, Writing – review & editing. **Xinyi Shou:** Supervision, Validation, Writing – review & editing. **Runzhe Su:** Validation, Writing – review & editing. **Jinyao Zhang:** Validation, Writing – review & editing. **Qiwen Hu:** Conceptualization. **Weilong Shang:** Methodology, Resources. **Xiancai Rao:** Funding acquisition, Writing – review & editing. **Renjie Zhou:** Funding acquisition, Writing – review & editing. **Xiao-Ran Jiang:** Conceptualization, Data curation, Writing – review & editing.

## Declaration of competing interest

The authors declare that they have no known competing financial interests or personal relationships that could have appeared to influence the work reported in this paper.
