# Supplementary material for: A user-friendly strategy to engineer tailored intermediate strains for overcoming combined type I and type II restriction-modification barriers in Staphylococcus aureus
Source: Synth Syst Biotechnol. 2026 Jul 3;16:13–23. doi: 10.1016/j.synbio.2026.05.016 (PMC13355661; doi:10.1016/j.synbio.2026.05.016)
Supplement: Multimedia component 1 [file mmc1.docx]

**A user-friendly strategy to engineer tailored intermediate strains for overcoming combined type I and type II restriction-modification barriers in *Staphylococcus aureus***

Yang Zheng ^a 1^, Chao Li ^a 1^, Xinqi Huang ^b^, Xinyi Shou ^b^, Runzhe Su ^b^, Jinyao Zhang ^b^, Qiwen Hu ^b^, Weilong Shang ^b^, Xiancai Rao ^b *^, Renjie Zhou ^a *^, Xiao-Ran Jiang ^b *^

^a^ Department of Emergency Medicine, the Second Affiliated Hospital of Army Medical University, Chongqing 400037, China

^b^ Key Laboratory of Microbial Engineering under the Educational Committee in Chongqing, Department of Microbiology, College of Basic Medical Sciences, Army Medical University, Chongqing 400038, China

* To whom correspondence should be addressed. Xiao-Ran Jiang Email: jiangxiaoran@tmmu.edu.cn. Correspondence may also be addressed to Xiancai Rao. Email: xcrao@tmmu.edu.cn. Renjie Zhou Email: zhourenjie@tmmu.edu.cn

^1^These authors contributed equally.


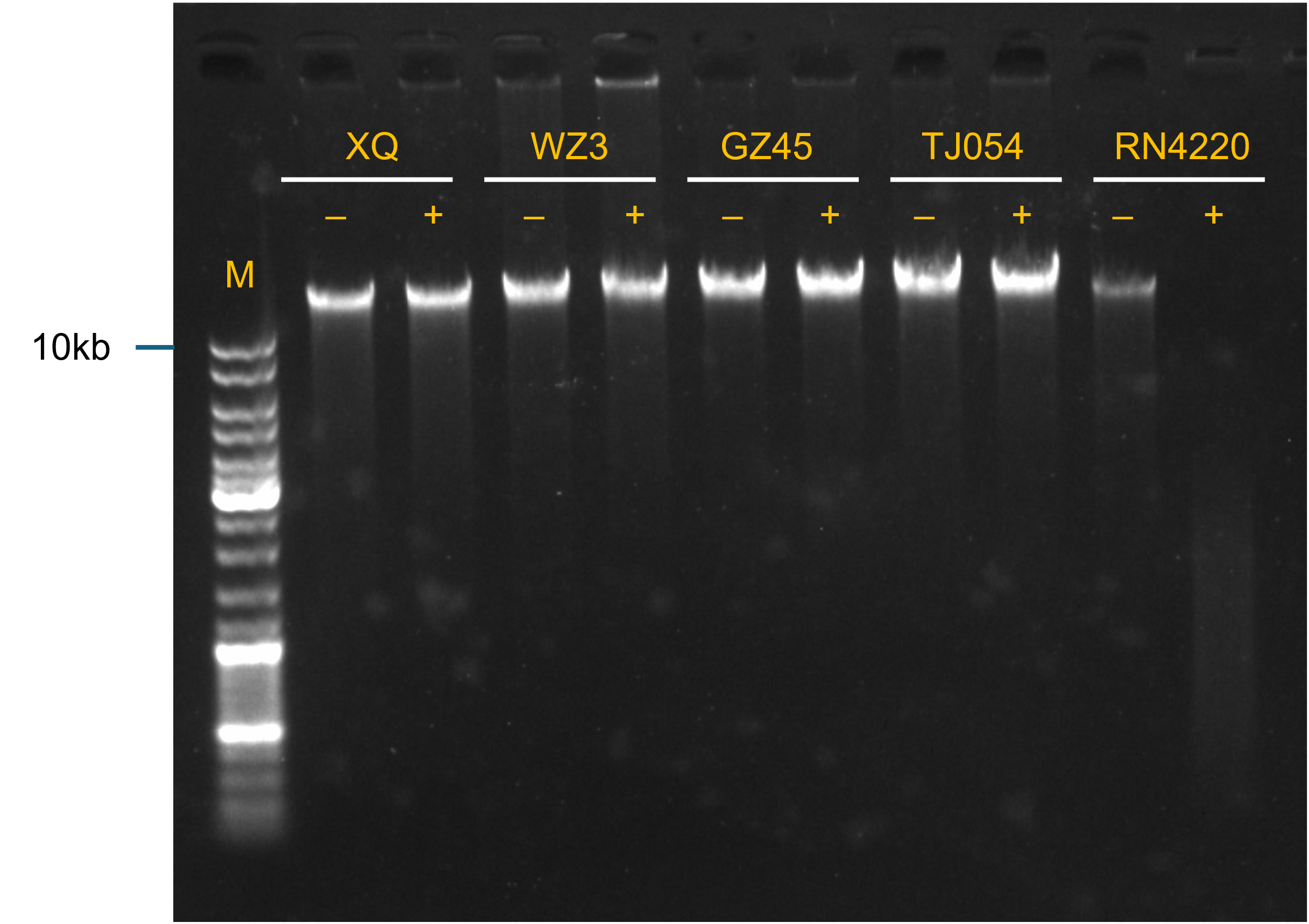


Figure S1. Sau3AI digestion assay of genomic DNA from *S. aureus* strains. Genomic DNA from four ST121 clinical isolates (XQ, WZ3, GZ45, TJ054) and the control strain RN4220 was either untreated (−) or digested (+) with Sau3AI restriction enzyme. Digestion reactions were carried out with 1 µg DNA and 5 U Sau3AI in NEBuffer r1.1 at 37 °C for 1 h. Each sample (100 ng DNA equivalent) was loaded onto a 1% agarose gel. All strains show high-molecular-weight genomic DNA (>10 kb). ST121 isolates (XQ, WZ3, GZ45, TJ054) display intact high-molecular-weight bands comigrating with undigested DNA, indicating resistance to Sau3AI digestion. RN4220 control shows complete degradation of genomic DNA after digestion, confirming susceptibility to Sau3AI.


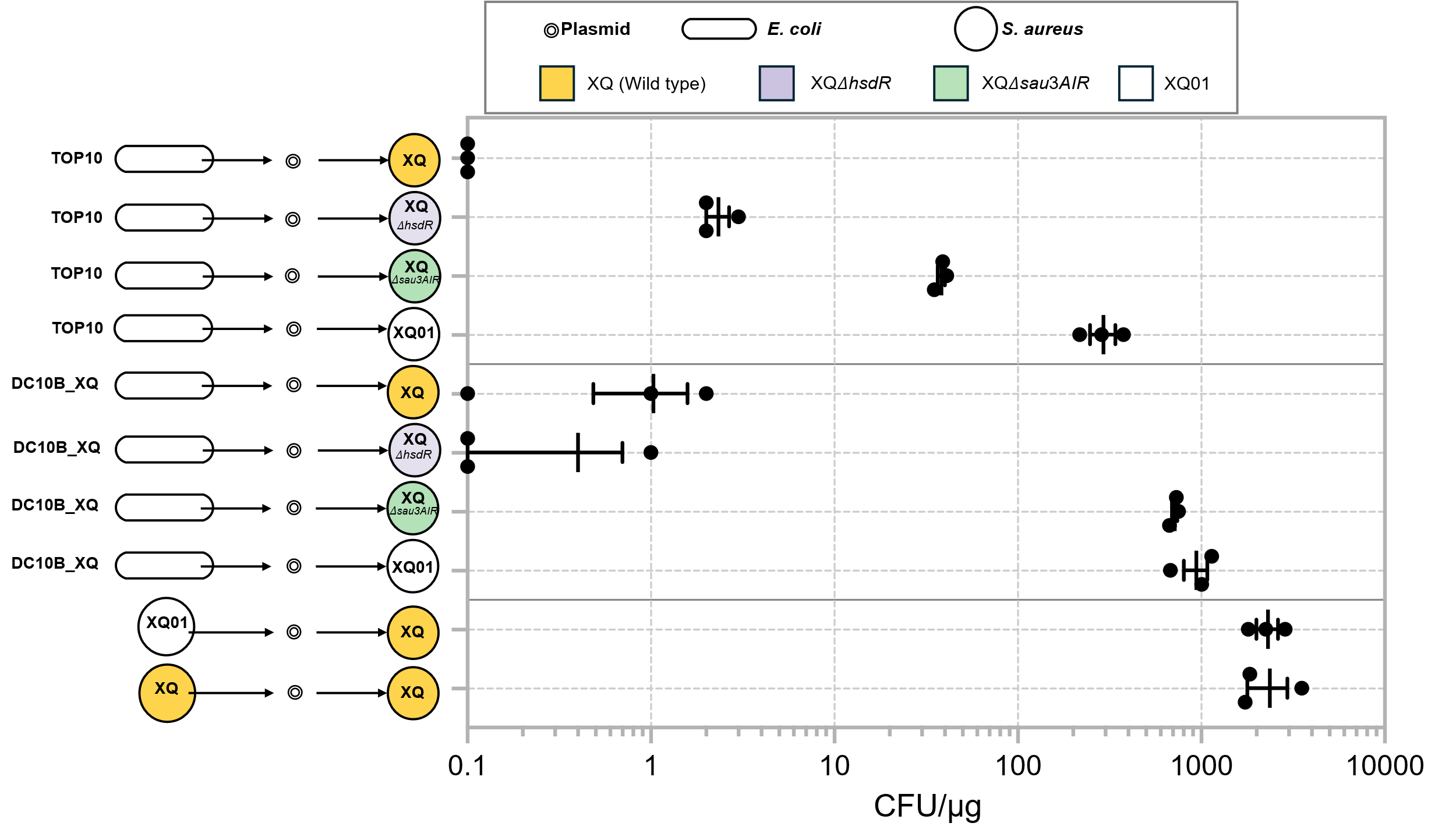


Figure S2. Electroporation efficiency of plasmids from different *E. coli* donors into *S. aureus* XQ and its restriction mutants under initial conditions (2.5 kV, 200 Ω, 25μF, 2 mm cuvette gap; recovery in BHI broth). Plasmids were isolated from three *E. coli* donor strains: the standard cloning host TOP10, the engineered intermediate strain DC10B_XQ (genomically integrated with the *hsdMS* modification subunits from *S. aureus* XQ), and the final donor strain XQ01. Recipient strains were wild-type *S. aureus* XQ and its derivatives: the type I restriction-deficient mutant (XQΔ*hsdR*), the type II restriction-deficient mutant (XQΔ*sau3AIR*), and the type I/II restriction-deficient double mutant XQ01. Individual data points from biological replicates are shown. Values of zero are plotted as 0.1 for logarithmic visualization. For direct comparison, the figure also includes results from plasmids extracted from XQ01 and from XQ itself electroporated back into XQ.


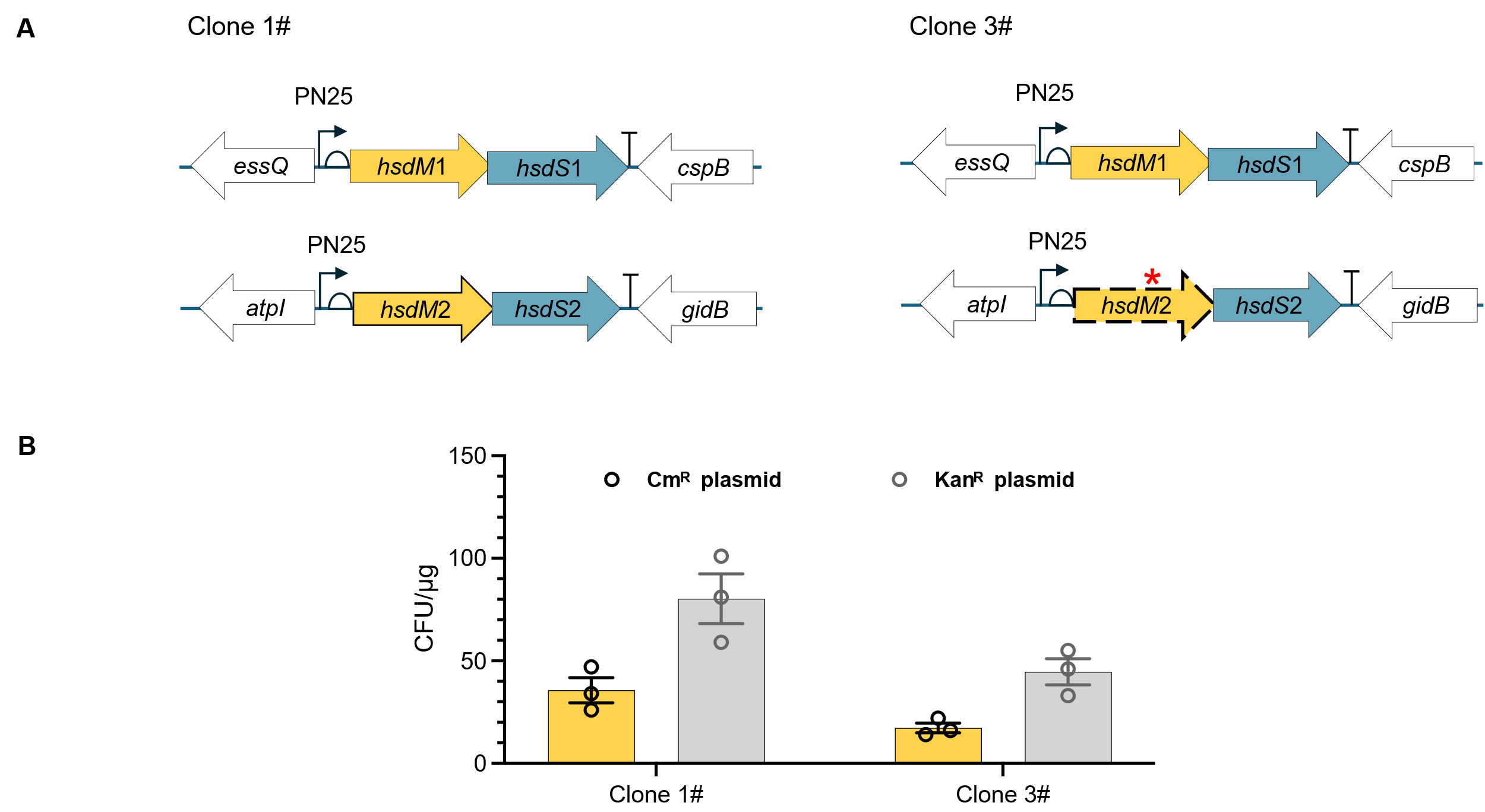


Figure S3. Validation and selection of the engineered intermediate *E. coli* host strain (DC10B_XQ). (A) Two candidate strains were obtained via CRISPR-Cas9 editing (as depicted in Fig. 1B): Clone 1# (desired genotype), Clone 3# (carrying a premature termination mutation in the *hsdM2* gene, indicated by a red asterisk). (B) Using the optimized electroporation conditions (2.1 kV, 200 Ω, 10μF, 1 mm cuvette gap; recovery in BHI supplemented with 0.5M sucrose) established in this study, two plasmids (SA24 and SA114) with different antibiotic resistance markers, each modified by the candidate strains, were electroporated into wild-type *S. aureus* XQ. Clone 1#, which conferred higher electroporation efficiency, was selected and designated as DC10B_XQ.


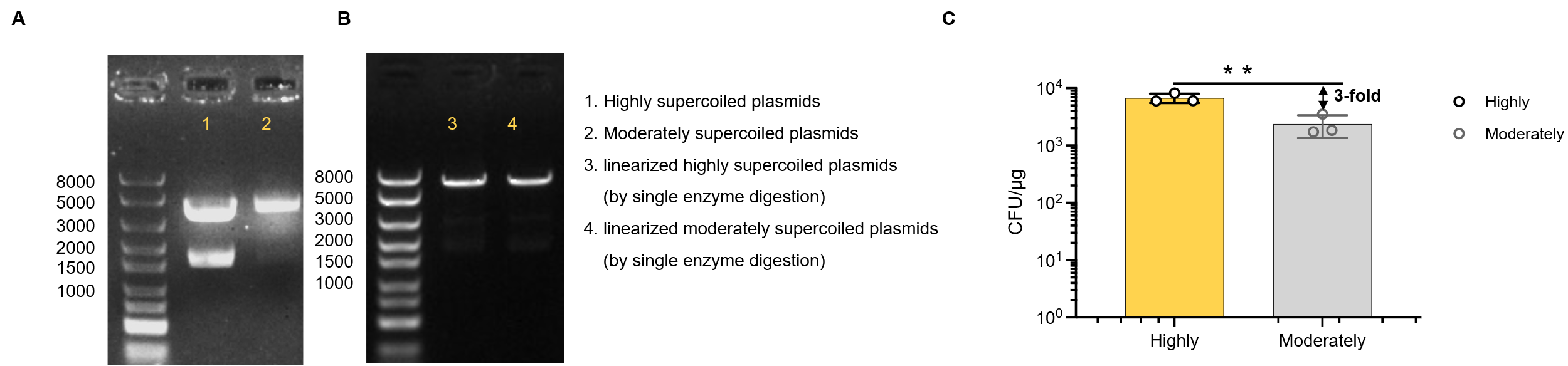


Figure S4. The degree of plasmid supercoiling influences transformation efficiency in *S. aureus* XQ. (A) Plasmids isolated from *S. aureus* XQ (6602 bp) showing different supercoiled forms on an agarose gel. The highly supercoiled form was freshly isolated, whereas the moderately supercoiled form was obtained after storage at 4 °C or -20 °C. (B) Comparison of highly and moderately supercoiled forms of the plasmid after digestion with KpnI-HF (single site). For digestion, 1 µg DNA was incubated with 10 units of enzyme in 1× CutSmartBuffer (NEB) at 37°C for 2 h; equal volumes of the reactions were loaded. (C) Transformation efficiency of *S. aureus* XQ with the plasmid of different supercoiling levels. Electroporation was performed under initial conditions (2 mm cuvette, 2.5 kV, 25 µF, 200 Ω; recovery in BHI broth only). Each symbol represents an independent biological replicate (n = 3). Data are mean ± SEM. **p < 0.01 by two‑tailed unpaired Student’s t‑test.

Table S1. Strains used in this study

| Strains | Description (relevant genotype or phenotype) | Source |
| --- | --- | --- |
| *E. coli* strains |  |  |
| TOP10 | *E. coli* (K-12) *dam* + *dcm* + *ΔhsdRMS* *endA1* *recA1* | commercial strain |
| DC10B | DH10B ∆*dcm*, does not methylate DNA on cytosine | Monk et al., 2015 |
| DC10B_XQ | DC10B with two XQ *hsdMS* clusters integrated in the *gidB–atpI* and *cspB–essQ* intergenic regions. | This study |
| *S. aureus* strains |  |  |
| RN4220 | ST8, a restriction-deficient derivative of *S. aureus* NCTC 8325 | A gift from Prof. Baolin Sun |
| USA300_FPR3757 | ST8, a widely used and sequenced representative isolate of the community-associated *S. aureus* USA300 lineage | ATCC BAA-1556 |
| XQ | ST121, a clinical *S. aureus* strain isolated from a patient with sepsis at Xinqiao Hospital, Chongqing, China | Lab stock  SAMN59651991 |
| XQW | XQ with a Q225P point mutation in the *sigB* gene | Lab stock |
| XQ∆*hsdR* | XQ with deletions of the *hsdR* gene | This study |
| XQ∆*sau3AIR* | XQ with deletions of the *sau3AIR* gene | This study |
| XQ01 | XQ with deletions of the *hsdR* and *sau3AIR* genes | This study |
| XQ∆*agrA* | XQ with a deletion of *agrA* gene | This study |
| XQ∆*hsdR::sfgfp* | XQ with *sfgfp* integrated in the *hsdR* locus | This study |
| XQWΔ*agrA eno-antares2* | XQW with deletion of *agrA* and *antares2* fused to the 3′ end of *eno.* | This study |
| WZ3 | ST121, a clinical *S. aureus* strain isolated from a hospital in Wenzhou, China | Lab collection  SAMN59651993 |
| GZ45 | ST121, a clinical *S. aureus* strain isolated from a hospital in Guangzhou, China | Lab collection  SAMN59651994 |
| TJ054 | ST121, a clinical *S. aureus* strain isolated from a hospital in Tianjin, China | Lab collection  SAMN59651992 |

Table S2. Plasmids used in this study

| Name | Description | Source / Accession number |
| --- | --- | --- |
| pCAS | repA101(Ts) ori, P_cas_-cas9 P_araB_-Red lacIq P_trc_-sgRNA-pMB1, Kanᴿ | Addgene #60847 |
| pTargetF | pMB1 ori, sgRNA, Spe^r^ | Addgene #62226 |
| pTargetF-ec | Derivative of pTargetF expressing a gRNA targeting the *essQ*-*cspB* intergenic region, without a donor template | This study |
| pTargetF-ag | Derivative of pTargetF with gRNA targeting the *atpI*-*gidB* intergenic region without donor editing template DNA | This study |
| SA12 | p15A ori(*E. coli*), RepF(*S. aureus*), PtetL-sfGFP, Kanᴿ, Cmᴿ(Sa) | This study  PZ366119 |
| SA13 | p15A ori(*E. coli*), RepRC (*S. aureus*), PtetL -sfGFP, Kanᴿ, Cmᴿ(Sa) | This study  PZ366120 |
| SA14 | p15A ori(*E. coli*), RepB(*S. aureus*), PtetL -sfGFP, Kanᴿ, Cmᴿ(Sa) | This study  PZ366121 |
| SA21 | p15A ori(*E. coli*), RepF(*S. aureus*), PXQ-arsR-sfGFP, Kanᴿ, Cmᴿ(Sa) | This study |
| SA22 | p15A ori(*E. coli*), RepF(*S. aureus*), PXQ-vraX-sfGFP, Kanᴿ, Cmᴿ(Sa) | This study |
| SA23 | p15A ori(*E. coli*), RepF(*S. aureus*), PXQ-ASU36_00490-sfGFP, Kanᴿ, Cmᴿ(Sa) | This study |
| SA24 | p15A ori(*E. coli*), RepF(*S. aureus*), PXQ-hu-sfGFP, Kanᴿ, Cmᴿ(Sa) | This study |
| SA25 | p15A ori(*E. coli*), RepF(*S. aureus*), PXQ_ASU36_08790-sfGFP, Kanᴿ, Cmᴿ(Sa) | This study |
| SA26 | p15A ori(*E. coli*), RepF(*S. aureus*), PXQ-amaP-sfGFP, Kanᴿ, Cmᴿ(Sa) | This study |
| SA27 | p15A ori(*E. coli*), RepF(*S. aureus*), PXQ-seb-sfGFP, Kanᴿ, Cmᴿ(Sa) | This study |
| SA28 | p15A ori(*E. coli*), RepF(*S. aureus*), PXQ-uspA-sfGFP, Kanᴿ, Cmᴿ(Sa) | This study |
| SA30 | p15A ori(*E. coli*), RepCts(*S. aureus*), PtetL -sfGFP, Kanᴿ, Cmᴿ(Sa) | This study  PZ366122 |
| pAEM_K | Shuttle vector with pMB1 ori (*E. coli*), RepF ori (S. aureus), PXQ-ASU36_00490-Kanᴿ | This study  PZ351570 |
| SA75 | Derivative of pAEM_K for allelic exchange to delete *agrA* in strain XQ | This study |
| SA77 | Derivative of pAEM_K for allelic exchange to delete *hsdR* in strain XQ | This study |
| SA92 | Derivative of pAEM_K for allelic exchange to fuse *antares2* to the 3' terminus of *eno* in strain XQ | This study |
| SA98 | Derivative of pAEM_K for allelic exchange to integrate *sfgfp* driven by promoter PXQ-arsR at the *hsdR* locus in strain XQ | This study |
| SA102 | Derivative of pAEM_K for allelic exchange to delete *sau3AIR* in strain XQ | This study |
| SA114 | pMB1 ori(*E. coli)*, RepF(*S. aureus*), PXQ-uspA-sfGFP, PXQ-ASU36_00490-Kanᴿ | This study |
| SA118 | p15A ori(*E. coli*), RepCts(*S. aureus*), PXQ-arsR-mCherry, PXQ-hu-Cmᴿ(Ec) | This study |
| SA120 | p15A ori(*E. coli)*, RepRC(*S. aureus*), PXQ-arsR-mCherry, PXQ-hu-Cmᴿ(Ec) | This study |

Cmᴿ(Sa): chloramphenicol resistance for selection in *S. aureus*, conferred by the cat gene from a shuttle vector backbone (e.g., pCasSA).

Cmᴿ(Ec): chloramphenicol resistance for selection in *E. coli*, conferred by the *cat* gene from transposon Tn9.

PXQ-[identifier] denotes the promoter region of the corresponding gene or locus tag from *S.aureus* strain XQ (e.g., PXQ-amaP for the *amaP* gene, PXQ-ASU36_00490 for the locus ASU36_00490)

Table S3. Sequences of promoters(containing intrinsic RBS) and representative genes

| PtetL |
| --- |
| cgttcaacaaacgggccatattgttgtataagtgatgaaatactgaatttaaaacttagtttatatgtggtaaaatgttttaatcaagtttaggaggaattaattatgaagtgtaatgaataatgaatgtaacagggttcaattaaaagagggaagcgtatcattaaccctataaactacgtctgccctcattattggagggtgaaat |
| PXQ-arsR |
| taatattttaataattatcattataacagtatttcttaaaaatgtaagatgtgttagaatatacgtataacttgctactaacaaagggattaataatcttttttataagtgtagaaacttatctgaaaaattaggtttttctcatttaattttaatgttagatagttataataaatagtaacgattaagaattttagaaaaaaatgttttttcttgatctataactaaattttaagcaatgaattttattcattcaaataggagagtgagatgt |
| PXQ-vraX |
| gattaaccacaccttatgatttgagtgtcattactgtgcgccagtaaagttagctttcaaagtctaactttgggtctcagtacacaataaagtcactttcggtgtggttattttagttattaaatacttgtttgattttcacagttaaattgggcaaataagtattgtaagtcatcattataagtagttttatttcaacatatgtatataacattcatttacagtgtcacgaatcagttatcctaaaaaaacaaatcctttcggaactttgttatgcccatgtttaaacataacgttttatcccgctctactaattctgccatttttttgaaattaaaaataacttctcaacgactttataaaaatatattccttttaaaacatgatacataaatagtcttagtaaatcatacgttctatgtctgataaaaagttgatacttaaggttgatgtcacacataaaaatgttatttataatataaccatagagtagcaaaggaggtaatataggtt |
| PXQ-ASU36_00490 |
| tgataagtgaaatataaaagtagtaattattatttgtaatataattgtaatatgactgttgttttagaaatgattgttgtataatggttgtgagcacaagacgatataaataaaaggagagagtgctt |
| PXQ-hu |
| aatgtgtattattacacatatacagctaataattacgcaaaatgaatagaaatggcttaaattcaacgtttttgccacataaatgattgcgatttatgtaatgcttgtgttaaagttttaccataatgatataaacatatcattatcaaacctcaggaggtgaatgtcta |
| PXQ_ASU36_08790 |
| ttcgatgataacggaattgacttacaaattagtaacatgaaaatttcaacgaaagacacaatgggcaaaatgttttttacgatgatgagtgcatttttcaggattagaagttaatttactatgtgagtgtataaaatagacttagcagcaacaagagcgagaggccgaaaaagcgggcgcccctctttaccagagaataaaaaatgagaaattaaatttttatatgatgaacaaacgataacaggggaagaaatagctagttagacaagggtatgtcactcaactgtttatcgagttgattaagaaatgaaaaaacttatactatgaattactgtttaaaagtgtgcatgttataatatttattgagcaagttggatagatggtggctaatctcttaataaaggggtgatgcctatggttatagttgttactcctagaaaggactagc |
| PXQ-amaP |
| ggagattgaagcaacgtttaattgtgctatatagtgttcataacattatacagtcataaaattagaactaggtagaaaaataattatgaaaaaacactatgttgttagagatgtttttgtatataagtttaggtgatgtatagttgtttaattttaaaagaataacttaattaatataaataacgtgagattattaaatataattaagtttcttttaatgttttttaattgaatatttaagattataacatatatttaaagtgtatctagatactttttgggaatgttggataaaggagataaaaa |
| PXQ-seb |
| ttaaaaattaggtttcattaatatgaactagggaatatattactaaatggtacttcatcttatatattaaaaataaatttatagttattaatttattatgtttaagaaaaaatctgatgggtacatatttaatatgtttttaaattaactttaactaaggaggtgtcgttaggg |
| PXQ-uspA |
| cgcgaacattttgaaagcgcaatcaaaaatccacaaaattgtaaaggttattacactgacttttccgaaaattgtggtaaaatataattaagaaagaacaaggaggcacttact |
| Cmᴿ(Ec) |
| atggagaaaaaaatcactggatataccaccgttgatatatcccaatggcatcgtaaagaacattttgaggcatttcagtcagttgctcaatgtacctataaccagaccgttcagctggatattacggcctttttaaagaccgtaaagaaaaataagcacaagttttatccggcctttattcacattcttgcccgcctgatgaatgctcatccggaatttcgtatggcaatgaaagacggtgagctggtgatatgggatagtgttcacccttgttacaccgttttccatgagcaaactgaaacgttttcatcgctctggagtgaataccacgacgatttccggcagtttctacacatatattcgcaagatgtggcgtgttacggtgaaaacctggcctatttccctaaagggtttattgagaatatgtttttcgtctcagccaatccctgggtgagtttcaccagttttgatttaaacgtggccaatatggacaacttcttcgcccccgttttcaccatgggcaaatattatacgcaaggcgacaaggtgctgatgccgctggcgattcaggttcatcatgccgtttgtgatggcttccatgtcggcagaatgcttaatgaattacaacagtactgcgatgagtggcagggcggggcgtaa |
| Cmᴿ(Sa) |
| atgaaatttaataaaattgatttagacaattggaagagaaaagagatatttaatcattatttgaaccaacaaacgacttttagtataaccacagaaattgatattagtgttttataccgaaacataaaacaagaaggatataaattttaccctgcatttattttcttagtgacaagggtgataaactcaaatacagcttttagaactggttacaatagcgacggagagttaggttattgggataagttagagccactttatacaatttttgatggtgtatctaaaacattctctggtatttggactcctgtaaagaatgacttcaaagagttttatgatttatacctttctgatgtagagaaatataatggttcggggaaattgtttcccaaaacacctatacctgaaaatgctttttctctttctattattccatggacttcatttactgggtttaacttaaatatcaataataatagtaattaccttctacccattattacagcaggaaaattcattaataaaggtaattcaatatatttaccgctatctttacaggtacatcattctgtttgtgatggttatcatgcaggattgtttatgaactctattcaggaattgtcagataggcctaatgactggcttttataa |

Table S4. Sequence of PN25-RBS-T7TE

| Sequences ( 5’ → 3’) |
| --- |
| cataaaaaatttatttgctttcaggaaaatttttctgtataatagattcataaatttgagatggttcgaagcggtccgtaggggggtaaataacatctgctaacaaagcccgaaaggaagctgagttggctgctgccaccgctgagcaataactagcataaccccttggggcctctaaacgggtcttgaggggttttttgctgaaaggaggaactatatccggat |

Table S5. Key primers and probes used in this study

| Sequences | 5’ → 3’ |
| --- | --- |
| atpI_F | gtcgcaattgtatgcactgg |
| atpI_R | tggatctatcaacaggagtcacgatcagcggcaagaatac |
| gidB_F | ctttttacggttcctggccttttgaccgtatctgcaaggtgaac |
| gidB-R | cctggttgttgttaacagtc |
| cspB_F | ctttttacggttcctggccttttgcggtttgaagaacagacgat |
| cspB_R | catgtattggacctccttac |
| essQ_F | tgcagtttgggcaggaacat |
| essQ_R | tggatctatcaacaggagtcttactctcctctcgcagcct |
| hsdMS1_F | ccgtaggggggtaaataacatatgtctattactgaaaaacaacgtcag |
| hsdMS1_R | ctttcgggctttgttagcagttaaacaaacattttttgtagaagcgattg |
| hsdMS2_F | ccgtaggggggtaaataacatatgtctattactgaaaaacaacgtca |
| hsdMS2_R | ctttcgggctttgttagcagccacttattaatcgagcagtaaaac |
| T7TE_F | ctgctaacaaagcccgaaag |
| RBS_R | atgttatttacccccctacgg |
| Inverse_F | cggttacttcattgaaccgc |
| Inverse_R | gcggttcaatgaagtaaccg |
| ΔargA_up_F | aactcggatgaagctaaagt |
| ΔargA_up_R | ggtaagttcactgtgactcg |
| ΔargA_dn_F | cgagtcacagtgaacttacctagaaactgcacatacacgcttac |
| ΔargA_dn_R | tacctttttgccattggcgc |
| ΔhsdR_up_F | tgagatctgtccatacccatcaccattcaagtccctccat |
| ΔhsdR_up_R | ctgtctcagtcgcttgttga |
| ΔhsdR_dn_F | tcaacaagcgactgagacagatggacgcttatcgtgagct |
| ΔhsdR_dn_R | aacttattaactctttccgctatccatagctacgcacctc |
| PXQ-arsR-sfGFP_F | ccttatgaggtgcttgagcaatcggaacgatcgttggctg |
| PXQ-arsR-sfGFP_R | atggcgattgacgacaaagctggcaattccgacgtctcat |
| Δsau3AIR_up_F | tgagatctgtccatacccatacgacttgcaagctcagatg |
| Δsau3AIR_up_R | atggcgttgccttaagttcc |
| Δsau3AIR_dn_F | ggaacttaaggcaacgccatcattagaagctgtaccggac |
| Δsau3AIR_dn_R | actggccgtcgttttacaacaaagcttcggcaaacaaacc |
| eno_up_F | ttctttcctgcgttgtcgacatgatctcattagacggtactcc |
| eno_ up _R | tgctcaccattttatctaagttatagaatgatttgataccg |
| eno_dn_F | tggacgagctgtacaagtgatcattttaaacattaattactgccataattttagttg |
| eno_dn_F | actggccgtcgttttacaacactgcttttaccttcttggag |
| antares2_F | cattctataacttagataaaatggtgagcaagggcgag |
| antares2_R | tcacttgtacagctcgtcca |
| sfgfp-F | TGAAGGTGACGCAACTAATGG |
| sfgfp-R | TAACGAGCAAAGCACTGAACAC |
| sfgfp-P | FAM-TACTACTGGTAAACTGC-MGB |
| nuc-F | TAAAGCGATTGATGGTGATACG |
| nuc-R | TTAGGATGCTTTGTTTCAGGTGT |
| nuc-P | VIC-ACAAAGGTCAACCAAT-MGB |

Table S6. The donor DNA for editing

| PN25-XQ *hsdMS1* |
| --- |
| ttactctcctctcgcagccttacgcctgtcttctttaatcttgaaataaagatttgtcagatacgtcagcaggccaaaaaccaggctacccagcacaccgattgcagcccactgtgacggagttactttatcgagtaactgcaatgcccagaaaccagcattacccgccgatgtgccataggcaacacctgttgttaacttatccattgatttcatatcctcaccccgatgtacacggatggtgcaatatgtttgaaaagatcggagtctacggggtagttttgacagcacacgttgttctcaacggcgctaaaaaaacatacacattaaaaatgtgggtaattattttgaaagaaagtcatatataaaataataatacgagaaatgttttcatatttagtgtactgtatacggccatttatacaggaaaagcctatgtcagaacgtaaaaactcaaaatcacgccgtaattatctcgttaaatgttcctgcccaaactgcacataaaaaatttatttgctttcaggaaaatttttctgtataatagattcataaatttgagatggttcgaagcggtccgtaggggggtaaataacatatgtctattactgaaaaacaacgtcagcaacaagctgaattacataaaaaattatggtcgattgcgaatgatttaagagggaaCatggatgcgagtgaattccgtaattacattttaggcttgattttctatcgcttcttatctgaaaaagcagaacaagaatatgcagatgccttgtcaggtgaagacatcacgtatcaagaagcatgggcagatgaagaatatcgtgaagacttaaaagcagaattaattgatcaagtcggttacttcattgaaccgcaagatttattcagtgctatgattcgtgaaattgaaacgcaagatttcgatatcgaacatctggcgacggcaattcgtaaagttgaaacatcaacactaggtgaagaaagtgaaaatgactttatcggactatttagcgatatggatttgagttcaacgcgTttaggtaacaatgtcaaagaacgtactgcgttaatttccaaagtcatggttaaccttgacgacttaccattcgttcacagtgacatggaaattgatatgttaggtgatgcatacgaattcctgattgggcgctttgcggcgacagcgggtaaaaaagcaggtgagttctatacaccacaacaagtatctaagatactggcgaagattgtgacagatggtaaagataaattacgtcatgtgtatgacccaacatgtggttccggttcattattgttacgtgtaggtaaagaaacgcaagtgtatcgttatttcggtcaagaacgtaacaatactacatataacttagcgcgcatgaacatgttattacatgatgtgcgttatgaaaatttcgatatccgtaatgatgacacgttggaaaatccagcctttttaggcaatacatttgatgcggttattgcgaacccaccatacagtgcgaaatggacagcagattcaaaatttgaaaatgatgaacgattcagcggttacggcaagcttgcgccaaaatccaaagcagactttgcctttattcaacacatggtacattacctagacgatgaaggtaccatggccgttgtactcccacatggtgtcttattccgtggtgccgcagaaggtgtcattcgtcgttatttaatcgaagaaaagaactacttagaagccgtgattgggttaccagcaaacattttctatgggacaagtattccaacatgtattttagtatttaaaaaatgtcgccaacaagacgacaacgtattatttatcgatgcatccaatgattttgaaaaaggaaaaaatcaaaaccatttaagcgatgcccaagtcgaacgcattattaacacatataaacgtaaagaaacgattgataaatatagctacagtgcgacattacaagagattgccgataacgattacaacttaaatataccgagatatgttgatacattcgaagaagaagcaccgattgatttagatcaagtccaacaagatttgaaaaatatcgacaaagaaattgcagaaattgaacaagaaatcaatgcatacctgaaagaacttggggtgttgaaagatgagtaatacacaaacgaaaaatgtgccagagttgagattcccagggtttgaaggtgaatattctttagacatttttggaaatctagcaacgaataagagtgataaatttaaccctcaaaatgagaatgcaagtattgatatagaattggattgtattgaacaaaatacgggtcgattaattaaaatttataattcaaaagaattttcaagtcaaaaaaataaattcaatccacaaaatgttttgtatgggaagctcagaccatatttgaataagtattattttacaaaaaaaagtggagtgtgttcatcagaaatatgggttttgaaatcaacgaaagaagataaattattgaatttatttctatattattttatacaaacaaaacgatattctgatgttgctagtaaatcggctgggtctaagatgccaagggctgattggggtttaatagaaaatataagagtatattttccagaattatgtgaacagcaaaaaataggcgaattcttcagcaaactcgaccgacaaattgtattagaagaacaaaaacttgaattacttcaacaacagaaaaaaggctatatgcagaaaattttctcacaggaattgcgattcaaggatgagaatggtaatgattatccggagtgggttgttaagttaataggtgatattggaaaagttgctatgaataaacgaatttataagcatgaaacttcagcaaatgaagaaatacccttttataaaataggtaattttggaaaaaaagcagatacatttatttctagagagcaatttaatgaatataaagaaaaatatccatatcctaaaaaaggtgacatcttaatttcggcttctggtagtattggaaaaacaattgaatacaaaggggaagaagcttattatcaagattcaaatatagtttggttagatcataatgaagaagtacttaatgtttttttaaagtatttttatattcttgttaaatggaatggagttgaaggaacaacaattaaaagactctataataaaaatattttgaatacaaaaatagacttaccaactattagtgaacagcaaaaaataggtgactttttttgctatattgataacttaattgaaatacaggaacaaaaacttgaatttttaaaacggagaaaacaatcgcttctacaaaaaatgtttgtttaactgctaacaaagcccgaaaggaagctgagttggctgctgccaccgctgagcaataactagcataaccccttggggcctctaaacgggtcttgaggggttttttgctgaaaggaggaactatatccggatcatgtattggacctccttactttttattaaagagatccaatattcactactctgtccgtatctctactcaggcatcagccttcttcgttatcgtatacagacgagcgatgaattttaatcagtaatgatgacatttgctgctgcaggacctttagcaccactctctatagagaaggtaaccttttgaccttcaaataaggttcgataattatcattctgaatcgcagaaaaatgcacaaacacatctttactaccatcaacaggagaaataaagccgaaacctttatcagcgttaaaccattttactaaaccagtcattttatttgacattctacattccttaacttgagcctttcggcataaatggtttgcataacagaaacgacttcgtacttaattggagagactcaaagaaggaataagtgaataacacctgaaatgagaactgctttagtaaactacttcgtatatcgtctgttcttcaaaccg |
| PN25-XQ *hsdMS2* |
| acgatcagcggcaagaataccgcctttaaaaccgccaacgccaccaccagtaacaccaacatcgccagaactttgaaagcttcgccaaatgcgaatgtccaggccacccggcctttcgctggtgtatgcgcctggtgacgccaggcaaatatcataaacaaaacgttaggcagaaagactgccaggcccccgcttattgcagagacgccccagaaggggtctttgaggctgaacagcaatccacttgctatcaccaccagtaactgaacgagcagaagcttccgagcaacgtttcgactcacgagcgacacagacatcacgtttttcactcctgctcccttcgaggtatgccgcgtgtcgtataaaactttctttaaggcttagagtcaagcatcaaaaagcggtcaaattatacggtgcgcccccgtgatttcaaacaataagtagccaaaaggtgaataaatgtttaaatatttttccagtgcatacaattgcgaccataaaaaatttatttgctttcaggaaaatttttctgtataatagattcataaatttgagatggttcgaagcggtccgtaggggggtaaataacatatgtctattactgaaaaacaacgtcagcaacaagctgaattacataaaaaattatggtcgattgcgaatgatttaagagggaatatggatgcgagtgaattccgtaattacattttaggcttgattttctatcgcttcttatctgaaaaagcggaacaagaatacgcagatgccttgtcaggtgaagaaatcacgtatcaagaagcatgggcagatgaagaatatcgtgaagacttaaaagcagaattaattgatcaagtcggttacttcattgaaccgcaagatttattcagtgcgatgattcgtgaaattgaaacgcaagatttcgatatcgaacatctggcgacggcaattcgtaaagttgaaacatcaacactaggtgaagaaagtgaaaatgactttatcggactattcagcgatatggatttgagttcaacgcgtttaggtaacaatgtcaaagaacgtactgcgttaatttccaaagttatggttaatcttgacgacttaccattcgttcacagtgacatggaaattgatatgttaggtgatgcatacgaatttcttatcgggcgctttgcggcgacagcgggtaaaaaagcaggcgagttctatacaccacaacaagtatctaagatactggcgaagattgtgacagatggtaaagataaattacgtcacgtgtatgacccaacatgtgggtcaggttcattactgttacgtgtaggtaaagaaacgcaagtgtatcgttatttcggacaagaacgtaacaataccacatacaacttagcacgcatgaacatgttattacatgatgtacgttatgaaaatttcgatatacgtaatgatgacacgttggaaaatccagcctttttaggccatacatttgatgcggttattgcgaacccgccatacagtgcgaaatggacagcagattcaaaatttgaaaatgatgaacgatttagtggttacggcaagcttgcgccaaaatccaaagcagactttgcctttattcaacacatggtacattacttagacgatgaaggtaccatggccgttgtactcccacatggtgtcttattccgtggtgccgctgaaggtgtcattcgtcgttatttaatcgaagaaaagaactacctagaagccgtgattggcttaccagccaatattttctatgggacaagtattccaacatgtattttagtgtttaaaaaatgtcgccaacaagatgacaatgtattatttatcgatgcatccaatgattttgaaaaaggaaaaaatcaaaaccatttaagcgacgcccaagtcgaacgcattatagacacatataagcgtaaggaaacaattgataaatatagctacagcgcgacattacaagaaatcgccgataacgattacaacttaaacataccgagatatgttgatacattcgaagaagaagcgccgattgatttagatcaagtccaacaagatttgaaaaatatcgataaagaaatcgcagaaattgaacaagaaatcaatgcatacctgaaagaacttggggtgttgaaagatgagtaatacacaaacgaaaaatgtgccagagttgagattcccagggtttgaaggcgaatgggaagagaagaagttagaagatattataaaagttaattctggaaaagattataaacatttggataaaggcgatataccagtctatggtactggcggttatatgacaagtgtttcagaaccactaagtgaaattgatgctgttggtattgggagaaaagggactataaacaaaccatatttgcttgaggcgccgttttggacggtggatacattattttattgtacacctaaaaaagaaacagacatactatttatattaagtttatttagaaaaataaattggaaagtatacgatgaatcaacaggtgtgccaagcttaagtaaacaaaccattaataaaataaatagatttgtccctacaaataaagagcagcaaaaaataggcaagttcttcagcaaacttgaccgacaaattgaattagaagaacaaaaacttgagttatttcagcaacagaaaaaaggctatatgcagaaaatcttttcgcaagaattgcgattcaaagatgagagtggtaatgattatccagattgggaagagaaggaattaggggaagtagctgatagagtaataaggaaaaataaaaactttgaatcgaaaaagcctttaacaatatccggacagttaggtttaattgatcaaacagaatattttagtaaatcagtttcgtcgaaaaatctagaaaattatacactaataaagaatggagaattcgcgtataataaaagttattctaatggatacccattaggggctattaaaagattaactagatatgatagtggtgtattgtcctctttgtatatttgcttttctattaaaagtgaaatgtctaaagacttcatggaagcatattttgattcgacacactggtatagagaagtttcaggaattgcagttgagggtgcaagaaatcacggattattaaatatttctgtgaatgatttttttactattctaattaaatatccaagtttagaagagcagagaaaaataggtgacttcttcatcaaacttgaccgacaaattgaactagaagaacaaaaactagaattacttcaacaacgaaaaaaagccttacttaaatcgatgttaatttaaatctgtaagagtttattttataggaaatggtaatgttttactgctcgattaataagtggctgctaacaaagcccgaaaggaagctgagttggctgctgccaccgctgagcaataactagcataaccccttggggcctctaaacgggtcttgaggggttttttgctgaaaggaggaactatatccggatcctggttgttgttaacagtctaaccggtcaattttttatgatttttttgataaaaattaaattttatttgctttaatcaccaccagatgacgttcgccatccagggctggaacctgaagtttaaccactgattcgacctgatattcttcgggcaacaaagcgatttcatcttccggcatttgccctttcagcgcgtagaaacggccttgctcaccaggaagatggtggcaccagctcaccatatcgttcagagaggcaaaagcgcggctaattacgccatcaaatggcggctctgaaggaaactcttctaccctgctctgtactggttcaatattctccagtttaagctcatgttgcacctgacgaaggaaacgcacgcgtttaccaaggctatccaacagagtgaaatgggcttcaggacgcacgatagagagtggaatgcctggcagtcctggtccggtgccgacatcgataaaccgttcaccttgcagatacgg |
| XQΔ*agrA* |
| aactcggatgaagctaaagtaataaggcagtattcttttatttttattggaatcactatatttttaagtatattaacatttgttatttcacaatttctccttaaagagatgaaatacaaaagaaatcaagaagaaattgagacctattacgaatacacattaaagattgaagcaattaataatgaaatgcgtaagttccgacatgattatgtcaatatcttaactacactttcagaatacattcgagaagatgacatgcctggtctacgtgactatttcaataaaaatatagtgccaatgaaagataacttacaaatgaacgctattaagttaaatggtattgagaatcttaaagtacgtgaaattaaaggcttaatcactgctaaaattttacgtgcacaagaaatgagtataccgattagtattgaaataccagatgaagtaactcacattaatttgaatatgattgatttaagtcgcagtattggtattattcttgataacgcaatagaggcatcaactgaaattgatgatcctatcattcgagttgcatttattgaaagcgaaaattcagtaacgtttattgttatgaataaatgtgcagatgatataccacgtattcatgaattgttccaagaaagtttttctactaaaggtgaaggtcgtggtttaggtctatcaactttaaaagaaattgctgataatgcagacaatgtcttattagatacaattatcgagaatggtttctttattcaaaaggttgaaattattaacaactaaccataaggatgtgaatgtatgaaaattttcatttgcgaagacgatccaaaacaaagagaaaacatgattaccattattaaaaattatataatgatagaagaaaagcctatggaaattgccctcgcaactgataatccttatgaggtgcttgagcaagctaaaaatatgaatgacataggctgttactttttagatattcaactttcaactgatattaatggtatcaaattaggcagtgaaattcgtaagcatgatccagttggtaacattatatttgttacgagtcacagtgaacttacctagaaactgcacatacacgcttacaattgttgtctaaagataatagcgttgaaacgattgaattaaaacgtggcagtaattcagtgtatgttcaatatgatgatattatgttttttgaatcatcaacaaaatctcacagactcattgcccatttagataaccgtcaaattgaattttatggtaatttaaaagaactgagtcaattagatgatcgtttctttagatgtcataatagctttgtcgtcaatcgccataatattgaatctatagattcaaaagagcgaattgtctattttaaaaataaagaacactgctatgcatcagtgagaaacgttaaaaaaatataataagataataaagtcagttaacggcgtattcaaatgcaaatcttgttggattttaacaagataactagcaaatgcactgtatagctggctttttaattttaataacaacactaaatatgacgcgtgaattaaaaaatgatgcaacgtttctttgtatacctattcctattactacataatttatcaatttctatttcactctacaatcctatggcgcaatttttaacaaaatgtactaaatttgaaatcgacagttttcttaaataaaatgctacaatttcctaccccgtgaatcttattttgttttaaaaaacagtgtagcacaagattcaacttgtttcctacactgttttataaatgaattatttaaaatgcttgttcaacctcttgtaatgttggcaaactattaatagcaccatacttcgttgtcactattgcagcaacgcgattgctaaacgctaaaatttcctcaccttcattttcaaataactgagttaaatttaacacatcggttgctaaaatcctgctaataactgcaccaataaaggcatcccctgccccagttgtatcaacaggttttaccttatagccactatgataatgattaataccatttttcaaatatacggcagcaccatctgctcccttcgtataaatgactacagtgacattacctgtaaataaagattgaatggcttcattttcatcatgaatacctgtaataaattctaattcttcatctgaaacttttacaatatgcgccaatggcaaaaaggta |
| XQΔ*hsdR* |
| tgagatctgtccatacccatcaccattcaagtccctccattaatcgtaggcaaaaaatttatcaatgatttgattatttgtattcaaattttagtatacgacttacctcaaacacaaatattaaaagataagacaataaaattagaataaatttacgtttgaagataaaaaacaaacatttttcattaaagtttatgatatatttagagcaatagaaagtgtacggaaggggatatgaatggcataccaaagtgaatacgcattagaaaatgaaatgatgaatcaacttgaacaattgggttacgaaagagtaacgatacgtgataataagcaattgcttgataattttagaacgattttaaatgagcgtcatgcggacaaattagaaggcaatcccttaacagataaagaatttcaacgtctgttaacgatgattgatgggaaaagtattttcgagagtgcccgtattttacgtgataaattaccacttagacgtgatgatgagtctgagatttatttgtcgtttttagatacgaaaagttggtgtaaaaataagtttcaagtgacgaatcaagtatctgtcgaggatacatataaagcacgttatgatgtaacgatattaatcaatggactaccccttgtccaagttgaattgaaacgtcgaggcattgatattaatgaggcgtttaaccaagtaaaacgttaccgtaaacaaaattacacaggcttattccgctacatacaaatgtttatcattagtaatggtgttgaaacgcgatacttttctaataatgatagcgaactattgaagagtcacatgttttattggagtgataaacagaataaccgaatcaatacattgcaatcgtttgctgagtcatttatgagaccttgtcaattagctaagatgatatcgcgctatatgattattaatgaaacagatagaatactgatggcaatgcgtccgtatcaagtgtatgcggtagaatcacttattcaacaagcgactgagacagatggacgcttatcgtgagcttaaaatgattgtgccgacaccacacatggttgatgacattcaagatgaagaagagctaaagcgctttgttgaagcttatcgtttattagctaaaataatattacgtttaaaagcatttgacgagtttgagtttacaattgatgaaattggaatggatgaacaagagaatgaagactataaaagtaaatatttagctgtgtacgatcaagtaaaaagagcgacggctgagaaaaacaaagtatccattttaaatgatattgatttcgaaatagaaatgatgcgtaatgatacgattaatgtgaattatattatgaatatattgagacaaattgatcttgaagacaaagcggaacaacgtcgtaaccaagaacaaattagacgtattttagatcatgcagatgatccgacattgaggttaaaacgagatctaattagagaattcatcgacaatgttgtaccttctttaaataaggatgatgatatcgatcaagaatatgttaatttcgaaagtattaaaaaagaagcggagttcaaaggatttgctggagagagatctatcgatgaacaagccctaaaaacaatttcaaatgactaccagtatagtggtgttgtaaacccacatcaccttaaaaaaatgattggtgatttgccattgaaagaaaaacgtaaagcaagaaaagccattgaatctttcgtggcagaaacaactgaaaaatacggtgtgtaatgattcagccccctcgctagattagtgtagggggcattattattttctatattttgttagttaccatgtgtttggcaatcatcatttggtttcaatacaattttatcatcgtatgtgtatagaaataatgtctataggcatttaaaatagtttataatattaacaggataagtattaaatgtatgttgaaggaggtgcgtagctatggatagcggaaagagttaataagtt |
| XQΔ*sau3AIR* |
| acgacttgcaagctcagatgcatcagtcgatagttgcgcgccatcagaaccatatactttaatgccattatagtctttcggattatgacttgctgtaatcataatgccagcagtagtattaagattgcgcaccgcgaacgataattccggtgtcgttttatatgtatcaggtaaataaactgttatttgatggtttgctaatacattagcaataatttgggcgaattcagttgaaagatgtctaatatcataatgaatgactattgttggactgtttgtttgggcatttaaataacgcgctaaacctaatgccaatttttcaatagtaaacttattaagtcgaccttcaccaagaccgaattttccgcgtatacccgcagtaccgaacgttaatttactttcaaaaccttctcgctgttcaatgtcagattgctgctcataaaaatgtttaactaaactatcattagctctttctatccataattctttatccattgttgctaaacatcctttcaaaatctcagttagacttaataaaacatgaaaactaaagcccttacatttatgtaatgaattataaagaaatacgccccaaaagtaaaaaaacacagccccgagacaatacttttcacaagtattatataatagatgtgtatgaaaatgcatggagtagatgtaagagtgatattcaaaatgtgtaaaaaatatggataattctatataattatattattgaaattttaaatagcggaaataattatttaatgttaagaggggataattttggaaagttatttgacaaaacaagccgtacataatcgcgcaaaagaagcagttggtaaaagcatattagaattaaatggtggtgaatcgattaaacaaagtaagagctcagttggtgatgcgtttgaaaattggtttggtaagaaaaaagacagtgatagtaaaccagatatggcagaagctggggtggaacttaaggcaacgccatcattagaagctgtaccggacaaaagtacaaaggatggttggcgaattaaaaataattttgtagataaaagtgatgatttaatatgccacgttagaccacacactaataacagagactatcgagggggaagtaatgcagataagcttcctaaaaagattaactggattaatagacctgactcagatgattattcggatgagtggatgactaagcaaagtttttggataaataatgactacattaaaatgcaagttgaagatttattgtagttaaagtatgttaaaatataagatatttctttcaaaatatcgaacgatcgttcgcttttatgttataataaggttgaattaattataggaggtcgcctaatgaataaaattaaagtagtagaattgtttgcgggtgtaggcgggtttcgtttaggtttagaaaatacgaaaaatggtatatttgacataacttgggcaaatcaatgggagccctcacgaaaaatccaacatgcatttgattgttatagtaaaagatttcagaacggcatccatagcaataaggatattgctgaggtatccgatgaagaaatggcaaatactgaagctgatatgattgtaggaggatttccttgtcaagattattcagttgcaaggagtttaaatggagaattaggaatacaagggaaaaagggcgttctattttggcaaattattagatatattcaaaatacatttcccaaatacttgttgcttgaaaatgtcgatagattattgaaatctccttcaagtcagagagggagagactttgctgtcatgttatcaactttaaatgagttaggctataatgttgaatggcgtgtgattaatgctgctgattatggtaatgctcaaagacgtagaagggtatttatatttggatataagcaagatttaaactatagtaaagctatggaagaaagtccgttggataaaataatatatcataatggtttgtttgccgaagcttt |
| XQ *eno-antares2* |
| atgatctcattagacggtactccaaacaaaggtaaattaggtgcaaatgctattttaggtgtatctatcgcagtagcacgtgcagcagctgacttattaggtcaaccactttacaaatatttaggtggatttaatggtaagcagttaccagtaccaatgatgaacatcgttaatggtggttctcactcagatgctccaattgcattccaagaattcatgattttacctgtaggtgctacaacgttcaaagaatcattacgttggggtactgaaattttccacaacttaaaatcaattttaagcaaacgtggtttagaaactgcagtaggtgacgaaggtggtttcgctcctaaatttgaaggtactgaagatgctgttgaaacaattatccaagcaatcgaagcagctggttacaaaccaggtgaagaagtattcttaggatttgactgtgcatcatcagaattctatgaaaatggtgtatatgactacagtaagttcgaaggcgaacacggtgcaaaacgtacagctgcagaacaagttgactacttagaacaattagtagacaaatatcctatcattacaattgaagacggtatggacgaaaacgactgggatggttggaaacaacttacagaacgtatcggtgaccgtgtacaattagtaggtgacgatttattcgtaacaaacactgaaattttagcaaaaggtattgaaaacggaattggtaactcaatcttaattaaagttaaccaaatcggtacattaactgaaacatttgatgcaatcgaaatggctcaaaaagctggttacacagcagtagtttctcaccgttcaggtgaaacagaagatacaacaattgctgatatcgctgttgctacaaacgctggtcaaattaaaactggttcattatcacgtactgaccgtattgctaaatacaatcaattattacgtatcgaagatgaattatttgaaactgctaaatatgacggtatcaaatcattctataacttagataaaatggtgagcaagggcgaggagctgatcaaggagaacatgagaagcaagctgtacctggaaggcagcgtgaacggccaccagttcaagtgcacccacgaaggggagggcaagccctacgagggcaagcagaccaacaggatcaaggtggtggagggaggccccctgccgttcgcattcgacatcctggccacccactttatgtacgggagcaaggtgttcatcaagtaccccgccgacctccccgattattttaagcagtccttccctgagggcttcacatgggagagagtcatggtgttcgaagacgggggcgtgctgaccgccacccaggacaccagcctccaggacggcgagctcatctacaacgtcaaggtcagaggggtgaacttcccagccaacggccccgtgatgcagaagaaaacactgggctgggagcccagcaccgagaccatgtaccccgctgacggcggcctggaaggcagatgcgacaaggccctgaagctcgtgggcgggggccacctgcacgtcaacttcaagaccacatacaagtccaagaaacccgtgaagatgcccggcgtccactacgtggaccgcagactggaaagaatcaaggaggccgacaacgagacctacgtcgagcagtacgagcacgctgtggccagatactccaacctgggcggaggcttcacgttagaagatttcgttggtgactggcgtcaaactgctggttataatttgtctcaagttttagaacagggtggtgtttcaagtttatttcaaaatttgggtgtcagtgttacgccaattcaacggattgttttaagtggtgaaaatggtttaaagatcgatattcatgtcatcattccatatgaaggtttatcaggtgaccaaatgggtcaaattgaaaaaattttcaaggtcgtttacccagttgataatcatcattttaaagttatcttgcactacggtacgttagttattgatggtgttacgccaaatatgattgattattttggtcggccatatgaaggcattgcagtttttgatggtaaaaagattacggttactggtacgttatggaatggtaataagattattgacgaacggttaattaacccagatggtagtttattattccgtgttacgattaatggtgttactggttggcgtttacacgaacgtattttagctaggcacgagctgatcaaggagaacatgagaagcaagctgtacctggaaggcagcgtgaacggccaccagttcaagtgcacccacgaaggggagggcaagccctacgagggcaagcagaccaacaggatcaaggtggtggagggaggccccctgccgttcgcattcgacatcctggccacccactttatgtacgggagcaaggtgttcatcaagtaccccgccgacctccccgattattttaagcagtccttccctgagggcttcacatgggagagagtcatggtgttcgaagacgggggcgtgctgaccgccacccaggacaccagcctccaggacggcgagctcatctacaacgtcaaggtcagaggggtgaacttcccagccaacggccccgtgatgcagaagaaaacactgggctgggagcccagcaccgagaccatgtaccccgctgacggcggcctggaaggcagatgcgacaaggccctgaagctcgtgggcgggggccacctgcacgtcaacttcaagaccacatacaagtccaagaaacccgtgaagatgcccggcgtccactacgtggaccgcagactggaaagaatcaaggaggccgacaacgagacctacgtcgagcagtacgagcacgctgtggccagatactccaacctgggcggaggcatggacgagctgtacaagtgatcattttaaacattaattactgccataattttagttgaggattattatgtcggtataaattgaataaagtttttgagttcacgcttaaaaagttcacgcttaaaaagttcacgcttgtatttataacctgccacagagttgagactgtggtaggttttttattttgaagtattattcataacagactaataatcatgaggtaactaataacacatatttaacttgtattcttaaactggtataataaatttatgttgaaatgaatattgtatgacagggtattcacttttattaaaaggtaaaaattaaataaaggttttatagaacgtatttaaatatatgaggagtaaacaaatggctgatagaacgaataaagaaattaaaacaggacgctttattgcaactgcatcaatcgtattctcaatattgttgattattcattactttgtttcgttggataatgcgactgccaaagcattactcaatttaacgaatcaaaacacttcagataaagcgatagattacattttaaacagctttagattcactggtattatgtatattttggcttatctagcaggcttcatcactttttggaatcgacatacttatgtgtggtggtttatgtttgcagtttatgtatcaaatagtttgtttacgttgattaatttatcaatcacaattcaagcaataaaagctgcacacggtgcgtacttaacattgccaattttaattgttattataggttcgattgcattagcgatttatatgcttgttgtttctatcaaacgtaaaagtacatttaatcgctagaaaattgattttaacaataaaaatatgatatactacttgtcgtatataaggaacggaggacaatttatgcatacatttttaatcgtattattaatcattgattgtattgcattaataactgttgtactactccaagaaggtaaaagcagtg |
| XQ *hsdR*::PXQ-arsR-*sfgfp* |
| tgagatctgtccatacccatcaccattcaagtccctccattaatcgtaggcaaaaaatttatcaatgatttgattatttgtattcaaattttagtatacgacttacctcaaacacaaatattaaaagataagacaataaaattagaataaatttacgtttgaagataaaaaacaaacatttttcattaaagtttatgatatatttagagcaatagaaagtgtacggaaggggatatgaatggcataccaaagtgaatacgcattagaaaatgaaatgatgaatcaacttgaacaattgggttacgaaagagtaacgatacgtgataataagcaattgcttgataattttagaacgattttaaatgagcgtcatgcggacaaattagaaggcaatcccttaacagataaagaatttcaacgtctgttaacgatgattgatgggaaaagtattttcgagagtgcccgtattttacgtgataaattaccacttagacgtgatgatgagtctgagatttatttgtcgtttttagatacgaaaagttggtgtaaaaataagtttcaagtgacgaatcaagtatctgtcgaggatacatataaagcacgttatgatgtaacgatattaatcaatggactaccccttgtccaagttgaattgaaacgtcgaggcattgatattaatgaggcgtttaaccaagtaaaacgttaccgtaaacaaaattacacaggcttattccgctacatacaaatgtttatcattagtaatggtgttgaaacgcgatacttttctaataatgatagcgaactattgaagagtcacatgttttattggagtgataaacagaataaccgaatcaatacattgcaatcgtttgctgagtcatttatgagaccttgtcaattagctaagatgatatcgcgctatatgattattaatgaaacagatagaatactgatggcaatgcgtccgtatcaagtgtatgcggtagaatcacttattcaacaagcgactgagacagccttatgaggtgcttgagcaatcggaacgatcgttggctgtaatattttaataattatcattataacagtatttcttaaaaatgtaagatgtgttagaatatacgtataacttgctactaacaaagggattaataatcttttttataagtgtagaaacttatctgaaaaattaggtttttctcatttaattttaatgttagatagttataataaatagtaacgattaagaattttagaaaaaaatgttttttcttgatctataactaaattttaagcaatgaattttattcattcaaataggagagtgagatgtatgcgtaaaggcgaagagctgttcactggtgtcgtccctattctggtggaactggatggtgatgtcaacggtcataagttttccgtgcgtggcgagggtgaaggtgacgcaactaatggtaaactgacgctgaagttcatctgtactactggtaaactgccggtaccttggccgactctggtaacgacgctgacttatggtgttcagtgctttgctcgttatccggaccatatgaagcagcatgacttcttcaagtccgccatgccggaaggctatgtgcaggaacgcacgatttcctttaaggatgacggcacgtacaaaacgcgtgcggaagtgaaatttgaaggcgataccctggtaaaccgcattgagctgaaaggcattgactttaaagaagacggcaatatcctgggccataagctggaatacaattttaacagccacaatgtttacatcaccgccgataaacaaaaaaatggcattaaagcgaattttaaaattcgccacaacgtggaggatggcagcgtgcagctggctgatcactaccagcaaaacactccaatcggtgatggtcctgttctgctgccagacaatcactatctgagcacgcaaagcgttctgtctaaagatccgaacgagaaacgcgatcatatggttctgctggagttcgtaaccgcagcgggcatcacgcatggtatggatgaactgtacaaataatgagacgtcggaattgccagctttgtcgtcaatcgccatatggacgcttatcgtgagcttaaaatgattgtgccgacaccacacatggttgatgacattcaagatgaagaagagctaaagcgctttgttgaagcttatcgtttattagctaaaataatattacgtttaaaagcatttgacgagtttgagtttacaattgatgaaattggaatggatgaacaagagaatgaagactataaaagtaaatatttagctgtgtacgatcaagtaaaaagagcgacggctgagaaaaacaaagtatccattttaaatgatattgatttcgaaatagaaatgatgcgtaatgatacgattaatgtgaattatattatgaatatattgagacaaattgatcttgaagacaaagcggaacaacgtcgtaaccaagaacaaattagacgtattttagatcatgcagatgatccgacattgaggttaaaacgagatctaattagagaattcatcgacaatgttgtaccttctttaaataaggatgatgatatcgatcaagaatatgttaatttcgaaagtattaaaaaagaagcggagttcaaaggatttgctggagagagatctatcgatgaacaagccctaaaaacaatttcaaatgactaccagtatagtggtgttgtaaacccacatcaccttaaaaaaatgattggtgatttgccattgaaagaaaaacgtaaagcaagaaaagccattgaatctttcgtggcagaaacaactgaaaaatacggtgtgtaatgattcagccccctcgctagattagtgtagggggcattattattttctatattttgttagttaccatgtgtttggcaatcatcatttggtttcaatacaattttatcatcgtatgtgtatagaaataatgtctataggcatttaaaatagtttataatattaacaggataagtattaaatgtatgttgaaggaggtgcgtagctatggatagcggaaagagttaataagtt |

For all the DNA sequences listed in Supplementary Table S6, the sequences colored orange, blue and red represent the main promoters, RBS sequences, and terminators, respectively.
